# Exploring Moderators and Mediators of the Outcome of Group Cognitive Behavioural Therapy Compared With Group Schema Therapy for Social Anxiety Disorder and Comorbid Avoidant Personality Disorder

**DOI:** 10.1002/cpp.70148

**Published:** 2025-10-13

**Authors:** Astrid E. Baljé, Anja Greeven, Mathijs Deen, Anne E. van Giezen, Arnoud Arntz, Philip Spinhoven

**Affiliations:** ^1^ Department of Anxiety Disorders Psyq The Hague the Netherlands; ^2^ Institute of Psychology Leiden University Leiden the Netherlands; ^3^ Parnassia Group Academy Parnassia Psychiatric Institute The Hague the Netherlands; ^4^ Department of Clinical Psychology University of Amsterdam Amsterdam the Netherlands

**Keywords:** Mechanisms of change, Random‐intercept cross lagged panel design, Randomised controlled trial, Schema modes, Self‐esteem

## Abstract

**Background:**

Identifying moderators and mediators in a randomised controlled trial is important to improve treatment effectiveness and elucidate mechanisms of change. Putative moderating and mediating variables of treatment outcome of 30 weekly sessions of group cognitive behavioural therapy (GCBT) and group schema therapy (GST) were investigated in a sample of 154 patients with both social anxiety disorder (SAD) and avoidant personality disorder (AVPD). Significant improvements were realised in both modalities at 3 and 12 months after treatment. No significant differences between conditions were found.

**Objectives:**

The current study explored several demographic and clinical patient characteristics as putative moderators of reducing SAD symptoms, AVPD manifestations, and treatment attrition. Emotion regulation, self‐esteem, experiential avoidance and schema modes were considered as putative mediators of SAD symptoms.

**Methods:**

Baseline variables moderating treatment effects on SAD symptoms and AVPD manifestations were investigated by comparing multilevel models. Differential effects of moderators on attrition hazard were examined by Cox regression. To assess possible mediators (measured pre‐, mid‐ and post‐treatment) of the effect of GCBT versus GST on SAD symptoms, separate three‐wave random‐intercept cross‐lagged panel models were performed.

**Results:**

No moderators and mediators were identified. Self‐esteem, the average mode score and avoidant protector mode at mid‐treatment predicted social anxiety at the end of treatment irrespective of treatment condition, while an inverse relationship was ruled out.

**Conclusions:**

The moderator analyses indicated that the examined patient characteristics cannot inform treatment decisions for either GCBT or GST. Furthermore, the mediation analysis did not point to different underlying treatment processes between both modalities.

## Summary

- Treatment for social anxiety disorder and comorbid avoidant personality needs to be optimised.
- Therefore, we explored patient characteristics and processes that potentially impact the outcome of cognitive behavioural therapy and schema therapy.
- Moderators and mediators were not differentially related to treatment outcomes.
- Three putative mediators at midpoint predicted subsequent social anxiety reduction in both treatments.
- Self‐esteem, the avoidant protector mode, and the schema mode constellation warrant attention in treatment, as they may promote the effects of therapy.

## 1 Introduction

Patients with social anxiety disorder (SAD) experience a persistent fear of humiliation and negative judgement in social situations involving unfamiliar people, or when they are at risk of possible scrutiny (APA 2000; APA 2013). Patients with avoidant personality disorder (AVPD) exhibit a pervasive pattern, present in a variety of contexts, that is characterised by social inhibition, feelings of inadequacy, hypersensitivity to negative evaluations, fear of rejection and reluctance to take personal risks or try new activities (APA 2000; APA 2013; Eikenæs et al. 2006). AVPD involves a more severe and broader area of personality dysfunction than SAD (Eikenaes et al. 2013). Prevalence estimates for SAD range between 7% and 13% (Steinert et al. 2013) and for AVPD they cluster around 1.5%–2.5%. AVPD also occurs in the absence of SAD (Lampe and Malhi 2018). The estimated comorbidity range of AVPD in SAD ranges from 50% to 89% (Cox et al. 2009). Over the years, the difference between the two disorders is well debated, and also AVPD conceptualised as a form of severe SAD has been proposed (Lampe and Sunderland 2015). SAD and AVPD remain classified as qualitatively distinct disorders, with SAD as a symptom disorder and AVPD as a personality disorder (PD) (APA 2013). Research attention for AVPD has been limited and mainly stems from studies on SAD reporting outcomes of cognitive behavioural therapy (CBT) for SAD with and without AVPD (Lampe and Malhi 2018; Simonsen et al. 2019). Schema therapy (ST) was developed by Young et al. (2003) as an alternative to CBT for treating PDs. In a mixed PD sample, Bamelis et al. (2014) conducted a randomised controlled trial (RCT) comparing ST with clarification‐oriented therapy and treatment as usual (e.g., insight‐oriented therapy, CBT). ST was found to be superior in the recovery of PDs, also in the large AVPD subsample. Given the need for treatment studies for AVPD, both CBT and ST merit further investigation.

In a RCT comparing group cognitive behavioural therapy (GCBT) and group schema therapy (GST) in patients with SAD and comorbid AVPD, no differences in the reduction of severity of social anxiety (SA) and AVPD were found. Treatment retention in GST was significantly higher. Both interventions led to significant and substantial improvements. Intention‐to‐treat analyses showed that within‐group effect sizes for SA symptoms were large for both GST and GCBT at 3‐ and 12‐month follow‐up. Cohen's *d* was 1.16 at T3 and 1.50 at T5 for GCBT, and 1.07 at T3 and 1.14 at T5 for GST. For AVPD manifestations, these values were 0.88 and 1.10, respectively, for GCBT, and 0.48 and 0.82, respectively, for GST (Baljé et al. 2024). However, many patients did not fully recover, highlighting the need to optimise treatment. These findings are not unique. Central to mental care is the question of how to make psychotherapy more effective and better individually tailored (Cuijpers et al. 2019), leading to the interest in process‐oriented research (Kopf‐Beck et al. 2020). Improvements in treatment outcomes might be realised by understanding the processes that account for therapeutic change, in which the identification of moderators and mediators forms an important step (Kazdin 2007).

### 1.1 Treatment Processes in CBT and ST

An important therapeutic target in CBT is the disconfirmation of dysfunctional beliefs by exposure. Over time, the understanding of the working mechanism of CBT for anxiety has shifted from habituation of the fear response to the belief that exposure in vivo sets off a process of inhibition. New associations (no‐unconditioned stimulus: e.g., non‐rejection) are formed around the conditioned stimulus (e.g., social situations) and inhibit the old association of the feared unconditioned stimulus (e.g., humiliation; Craske et al. 2014). In CBT for SAD in our RCT, SA was considered a learned response to social situations with avoidance and safety‐seeking behaviours as important maintaining factors. By integrating cognitive restructuring and exposure, individuals were helped to overcome avoidance of anxiety‐provoking social situations and test the reality of dysfunctional beliefs (Heimberg and Becker 2002).

ST aims to treat pervasive, long‐term psychological difficulties less responsive to traditional cognitive therapy, such as PDs. It is assumed that traumatisation in childhood and frustration of basic childhood needs lead to the development of early maladaptive schemas (EMS) and dysfunctional schema modes (SMs), which cause psychological problems in adult life (Young et al. 2003). SMs are negative emotional–cognitive behavioural states (Fassbinder and Arntz 2021). It has been proposed that when an EMS is triggered, the individual's coping results in a related SM activation (Young et al. 2003). ST aims to help patients understand their emotional core needs and learn adaptive ways of getting their needs met. This requires breaking through long‐standing emotional, cognitive and behavioural patterns, meaning change of dysfunctional schemas, coping strategies and SMs. Research on mechanisms of change in ST is still in its infancy (Fassbinder and Arntz 2021; Yakin et al. 2020).

### 1.2 Putative Moderators

Patients with the same diagnosis may vary in important ways, resulting in different responses to treatment. Clinical and sociodemographic characteristics such as comorbidity (e.g., depressive disorder/symptoms), PD traits, unemployment and being single may affect a patient's response to treatment (Lutz et al. 2021). While predictors influence treatment response regardless of treatment conditions, moderators have a differential influence on treatment response depending on treatment assignment. Investigation of putative moderators may help to identify variables that can guide treatment decisions for individual patients (Huibers et al. 2015; Vousoura et al. 2021). Adverse childhood experiences are hypothesised to underlie the development of maladaptive schemas that might be at the core of PDs and chronic syndrome disorders (Young et al. 2003). Several studies show associations between childhood adversity and AVPD, such as emotional neglect (Johnson et al. 2000), low parental affection (Johnson et al. 2006) and sexual and emotional abuse (Lobbestael et al. 2010). The association between adversive childhood events and AVPD was recently confirmed by a meta‐analysis (Crișan et al. 2023).

### 1.3 Putative Mediators

#### 1.3.1 Schema‐modes (SMs)

There is a need for further research on the role of SMs in therapeutic change. Although ST addresses SMs, ST theory does not state that the role of SMs in the change process during treatment is unique to ST (Yakin et al. 2020). In an RCT including patients with AVPD (Bamelis et al. 2014) comparing ST, treatment as usual, and clarification‐oriented psychotherapy, reductions in the Vulnerable Child (VC) and the Avoidant Protector (AP) modes, and an increase in the Healthy Adult (HA) mode, preceded reductions in PD severity and improvements in functioning, irrespective of treatment. This led Yakin et al. (2020) to suggest that SMs can be considered a possible common mechanism of change for PDs. Relevant modes for AVPD are the AP, characterised by interpersonal and situational avoidance, found to correspond to AVPD in a study of conceptualisations for specific PDs (Bamelis et al. 2011). The happy child (HC) mode, a playful and spontaneous mode, was the most negatively correlated mode with AVPD (Lobbestael et al. 2008). Besides the HA, the AP and the HC modes, therefore, seem relevant for patients with SAD and AVPD.

#### 1.3.2 Self‐Esteem (SE)

SE refers to a person's global evaluation or liking of him/herself in affective terms (Rosenberg 1979). It affects transactions of people with their environment (Kernis 2003) and influences perceptions and coping behaviour (Mann et al. 2004). Individuals with low SE are reluctant to risk failure or rejection because they lack a positive feeling of self‐worth to protect them. They are more likely to employ self‐protective strategies (Zeigler‐Hill 2011). SE showed prognostic value for the recurrence of depression and anxiety (van Tuijl et al. 2020). Patients with both SAD and AVPD tend to report more personality dysfunction regarding SE than patients with SAD but without AVPD (Eikenaes et al. 2013). Compared with patients with borderline PD, patients with AVPD showed significantly lower SE, and AVPD contributed to lower SE beyond what could be explained by comorbid depression (Lynum et al. 2008). Low SE is considered a risk and maintenance factor for mental disorders (Barbalat et al. 2022). A review of changes in self‐related constructs during CBT for SAD, including three studies on SE, suggested that changes in these constructs may be a basis for individuals' reduction in SAD as a result of CBT (Gregory and Peters 2017). Treatment of SAD enhanced SE (Ritter et al. 2013; Salaberria and Echeburua 1998; Taylor et al. 1997), but to our knowledge, there are no formal mediation studies examining to what extent treatment‐induced changes in SE predict subsequent reductions in SA symptoms.

#### 1.3.3 Emotion Regulation (ER)

Emotion dysregulation is increasingly considered a transdiagnostic mechanism that contributes to, exacerbates and maintains mental illness (Goodman et al. 2021). ER involves adaptive ways of responding to emotional distress, including the awareness, understanding and acceptance of emotions, the ability to control impulsive behaviours and engage in goal‐directed behaviours when experiencing negative emotions, flexible use of situationally appropriate strategies to modulate the intensity and duration of emotional responses in order to meet individual goals and situational demands, and a willingness to experience negative emotions in pursuit of desired goals (Gratz et al. 2015; Gratz and Roemer 2004). The relative absence of any or all of these abilities indicates the presence of difficulties in ER (Gratz and Roemer 2004). Change in ER processes has been proposed as one key mechanism of action in CBT for mood and anxiety disorders (Campbell‐Sills et al. 2006; Goldin et al. 2014; Hofmann et al. 2012). In ST, problems in ER are seen as a consequence of early adverse experiences leading to unprocessed traumas and fear of emotions, resulting in attempts to avoid emotions. Addressing these problems is supposed to improve ER (Fassbinder et al. 2016).

#### 1.3.4 Experiential Avoidance (EA)

EA refers to the unwillingness to remain in contact with aversive private experiences (e.g., sensations, emotions, thoughts, memories) and attempts to escape from and avoid these experiences. Paradoxically, EA has been shown to increase the frequency of these experiences and the associated distress. EA is a psychological process proposed as an aetiological and maintenance factor (Hayes et al. 1996). There is empirical support for the association of EA with anxiety disorders, including SAD (e.g., Spinhoven et al. 2014). CBT significantly reduced EA across heterogeneous anxiety disorders, and changes in EA preceded and predicted changes in anxiety, but not vice versa, supporting EA as a transdiagnostic mechanism in CBT (Eustis et al. 2020). We are not aware of studies on EA and ST, or AVPD.

In conclusion, to the best of our knowledge, moderators and mediators of treatment have not yet been investigated in a sample of patients with SAD and comorbid AVPD. Therefore, the current study employed an exploratory approach aimed at investigating several putative candidates, thereby generating knowledge to inform the formulation of hypotheses for future studies (Kraemer et al. 2006). We included characteristics commonly examined as predictors of treatment outcome or attrition (Lutz et al. 2021), or presumably related to treatment processes in CBT and/or ST treatment. An exploration of moderators of our RCT results might reveal which patients would benefit more from either GCBT or GST, while detecting differential mediators might indicate different underlying mechanisms (Huibers et al. 2015; Kraemer et al. 2002). Our main research question was the following: Are there moderators and mediators of the effect of GST and CCBT? First, we explored whether baseline patient characteristics differentially moderated the effect of treatment on SAD symptoms, manifestations of AVPD and treatment attrition. Second, after examining the effect of treatment on putative mediators, namely ER, EA, SMs and SE, we examined whether these putative mediators mediated the effect of treatment on SAD symptoms.

## 2 Methods

### 2.1 Participants and Procedures

This study is based on an RCT comparing GCBT with GST both offered in a semi‐open group format of 30 weekly sessions offered to a patient sample with SAD and AVPD as principal DSM‐IV diagnoses. CBT was based on the group CBT protocol for SAD (Heimberg and Becker 2002) combined with recent insights on exposure (Craske et al. 2014). GST was based on the group treatment for borderline PD (Farrell et al. 2014; Farrell and Shaw 2012); see Baljé et al. (2016) and Baljé et al. (2024) for more detailed information on methods, treatments, supervision and treatment integrity.

### 2.2 Treatment Outcomes, Potential Predictors, Moderators and Mediators

The primary outcomes of the RCT were SA symptoms and manifestations of AVPD. These were measured by the Liebowitz Social Anxiety Scale (LSAS) and the Avoidant Personality Disorder Severity Index (AVPDSI), respectively. Furthermore, attrition due to patient dropout or protocol violation was registered. Baseline patient characteristics (see Table 1), including sociodemographic/clinical variables and questionnaires (ER, SE, EA, SMs), were explored as putative predictors and moderators of outcomes on the LSAS, AVPDSI and attrition. PDs generally respond more slowly to treatment than symptom disorders. Therefore, AVPD severity was not measured at mid‐treatment. Hence, mediation analyses were limited to the LSAS and included measurements at baseline (T0), mid‐treatment (T1) and post‐treatment (T2).

**TABLE 1:** Baseline characteristics of the GCBT and GST group of study participants.

	Characteristic	GCBT (*n* = 79)		GST (*n* = 75)	
		***n***	**%**	***n***	**%**
	***Sociodemographic variables***				
►	Education level				
	– Low	5	6.3	5	6.7
	– Medium	28	35.4	30	40.0
	– High	16	20.3	22	29.3
	– Advanced	30	38.0	18	24.0
►	Work or study—yes	52	65.8	42	56.0
►	Civil status married/cohabiting	14	17.7	17	22.7
	***Clinical variables***				
►	Medication at start treatment—yes	21	26.6	29	38.7
►	Psychological treatment last 3 years				
	– No treatment	27	34.2	18	24.0
	– 1–5 sessions	4	5.1	6	8.0
	– 6–10 sessions	6	7.6	8	10.7
	– 11–20 sessions	10	12.7	14	18.7
	– More than 20 sessions	32	40.5	29	38.7
►	Depressive disorder present at start treatment	32	40.5	35	46.7

		**Mean**	**SD**	**Mean**	**SD**
►	Total number of axis I disorders	2.3	1.3	2.4	1.1
	Traits SCID‐II (SCID‐IV‐P)				
►	– Avoidant PD	5.4	1.0	5.4	1.0
►	– Dependent PD	0.8	1.0	0.8	1.2
►	– Obsessive‐compulsive PD	0.8	1.1	0.9	1.2
►	– Borderline PD	0.5	0.8	0.7	1.1
	***Questionnaires***				
	Childhood Trauma Questionnaire (CTQ‐SF)				
►	– Emotional neglect	13.2	5.1	13.8	5.5
►	– Physical neglect	7.5	2.8	7.6	3.4
►	– Emotional abuse	10.3	4.8	11.3	5.8
►	– Physical abuse	6.3	2.5	6.3	2.8
►	– Sexual abuse	6.3	3.2	6.5	3.4
►	Inventory of depressive symptomatology (IDS‐SR)	31.6	12.1	32.6	12.2
►▲	Acceptance and Action Questionnaire (AAQ‐II)	35.5	9.2	33.5	8.9
►▲	Difficulties in Emotion Regulation Scale (DERS)	91.5	22.0	92.1	21.8
►▲	The Rosenberg Self‐Esteem Scale (RSES)	11.2	4.4	11.3	4.9
	Schema mode inventory (SMI‐2)				
►▲	– SMI (average score)	3.2	0.5	3.2	0.5
►▲	– SMI‐Avoidant protector	4.3	0.8	4.3	0.7
►▲	– SMI‐Health adult	3.0	0.6	3.0	0.7
►▲	– SMI‐Happy child	2.8	0.7	2.7	0.6

*Note:* Included in: ► predictor/moderator analyses, ▲ the mediator analyses.

Abbreviations: GCBT = Group cognitive therapy, GST = Group schema therapy, *n* = number, SD = standard deviation.

### 2.3 Measures

#### 2.3.1 Diagnostic Assessment and Main Outcome Measures

The presence of symptom disorders and PDs was assessed with the Mini‐International Neuropsychiatric Interview (MINI; Sheehan et al. 1998) and the Structured Clinical Interview for DSM‐IV Axis II Personality Disorders (SCID‐II; First et al. 1997), respectively. Primary outcomes of the RCT were SA symptoms, measured with the LSAS (Liebowitz 1987), and manifestations of avoidant PD, measured with the AVPDSI (Baljé et al. 2023). In addition, attrition due to patient dropout or protocol violation was registered.

#### 2.3.2 Putative Moderating Variables

The Childhood Trauma Questionnaire (CTQ; Bernstein et al. 2003) was used to measure forms of childhood trauma: emotional and physical neglect, and emotional, physical and sexual abuse. Severity of depressive symptoms was assessed with the Inventory of Depressive Symptomatology Self‐Report (IDS; Rush et al. 1996). For both questionnaires, higher (sub)scale scores represent worse outcomes. Depressive disorders and total of axis‐I disorders were assessed with the MINI, and PD traits with the SCID‐II.

#### 2.3.3 Putative Moderating and Mediating Variables

SE was measured with the Rosenberg Self‐Esteem Scale (RSES; Rosenberg 1965), a higher score presenting higher self‐esteem. EA was assessed with the Acceptance and Action Questionnaire (AAQ‐II; Bond et al. 2011), higher scores indicating more acceptance and less experiential avoidance. ER was measured with the Difficulties in Emotion Regulation Scale (DERS; Gratz and Roemer 2004). The awareness items were excluded to optimise its psychometric properties (Hallion et al. 2018). More difficulties in ER are represented by higher scores. To measure the strength of SMs, we used the Schema Mode Inventory‐2 (SMI‐2), which measures 18 SMs (Lobbestael et al. 2008). We added the HC mode from the SMI‐1 (SMI; Lobbestael et al. 2010) because of its putative therapeutic relevance in the ST for patients with AVPD. In order to reduce the number of analyses, a Principal Component Analysis (PCA) was performed. This revealed a one‐component model as a suitable and interpretable solution (see Supplementary Table A1). In further analyses, the average of the means of all SMI subscales, hereafter referred to as SMI‐AV, was therefore used. In addition, the AP, HA and HC subscales were also separately examined as possible predictors, moderators and mediators, since for patients with AVPD strengthening the healthy modes (HA, HC) and weakening the AP mode were important goals in GST. For the SMI‐AV and AP, higher scores are less favourable. For the HA and HC, higher scores are more favourable. Cronbach's *α* of measures, as found in the present study, are shown in Supplementary Table A2. See Baljé et al. (2023) for psychometric properties of the AVPDSI and Baljé et al. (2016) for all other measures.

### 2.4 Analyses

#### 2.4.1 Baseline Characteristics as Putative Moderating Variables

First, for the analysis regarding baseline patient characteristics and scores as potential predictors and moderators of differential treatment effects, all mean‐centred variables were separately added to the multilevel model containing an interaction between time and treatment.

For the LSAS, we encountered convergence problems in the optimisation procedure of the random effect model. Therefore, we incorporated a generalised least squares model with the explicit specification of the error covariance structure to properly account for dependence in the data (Jennrich and Schluchter 1986). A likelihood ratio test indicated a heterogeneous autoregressive structure (ARH) over a first‐order autoregressive structure (AR1). For each candidate predictor, likelihood ratio tests (LRT) were used to investigate whether a model with only a main effect, with an interaction between the candidate predictor and time, or with a three‐way interaction (time * condition * candidate predictor, moderation) had a better fit. If the LRT indicated a model with an interaction effect, the ANOVA and fixed effects were examined. Cox regression was used to explore which baseline patient characteristics and scores were significantly related to differential effects on time to attrition by investigating main effects and interaction effects with condition.

#### 2.4.2 Multivariate Analyses of Moderation of Baseline Characteristics

Second, best subset selection was performed to assess the potential interaction of baseline variables with time in a multivariate way in the prediction of LSAS and AVPDSI outcomes (James et al. 2021). All possible combinations of interaction effects (time * predictor) were investigated using the conservative Bayesian Information Criterion (BIC; Schwarz 1978) as the model selection criterion. Due to a potential risk of Type I error inflation caused by the preselection of variables (Sun et al. 1996), *p*‐values were not interpreted.

#### 2.4.3 Mediation Analyses

First, differential treatment effects on the DERS, AAQ, RSES and SMI were individually examined by multilevel models to determine if time effects significantly differed for GCBT and GST. Due to convergence problems in the optimisation procedure of the random effect model, we applied an error covariance structure directly (AR1 or ARH).

We used random intercept cross‐lagged panel models (RI‐CLPM) on an intent‐to‐treat basis to investigate mediation in Mplus Version 8.0. To evaluate model fit, we used both CFI and SRMR with cut‐off values close to 0.09 and 0.95, respectively (Hu and Bentler 1999; Shi et al. 2022). Missing data were handled by full information maximum likelihood estimations.

**FIGURE 1:**
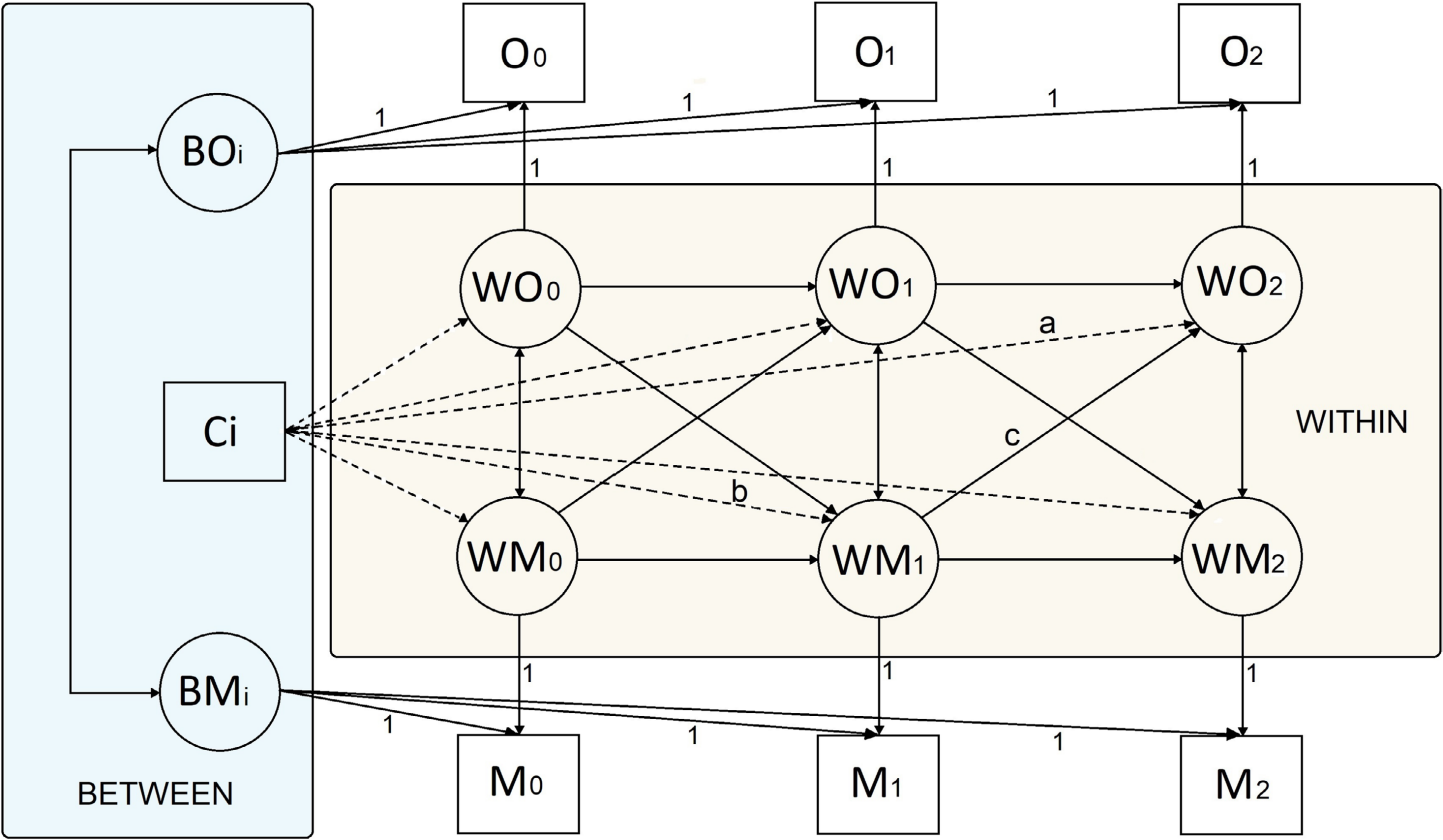
Random intercept cross‐lagged panel model of mediation of outcome at post‐treatment by mediator at mid‐treatment. Note: 0, 1, 2 is baseline (T0), mid‐treatment (T1) and end‐treatment (T2), respectively; O = outcome (LSAS), M = mediator, B = between, W = within; Ci = Condition, i.e., Group Schema therapy (GST) versus Group Cognitive Behavioural Therapy (GCBT). a = Path a, differential effect of condition on outcome T2; b = Path b, differential effect of condition on mediator; c = Path c, prediction of anxiety at T2 by mediator at T1.

RI‐CLPM is a structural equation modelling (SEM) approach to longitudinal data (see Figure 1 for a graphical presentation of the RI‐CLPM model). It decomposes data into stable trait‐like between‐person differences (B) and fluctuating within‐person differences (W). Trait‐like stability is captured by the random intercepts (B0i, BMi). Lagged relations pertain exclusively to within‐person fluctuations. This distinction is important as only intra‐individual associations can represent causal effects over time (Hamaker et al. 2015; Mulder and Hamaker 2021).

Each putative mediator (AAQ, DERS, RSES, SMI‐AV, SMI‐HA, SMI‐HC, SMI‐AP) was tested in a separate three‐wave model. The within‐components consisted of cross‐sectional associations between the outcome and mediator; autoregressive effects, i.e., within‐person carry‐over effects; and cross‐lagged effects, i.e., spill‐over of the state between the mediator and outcome at subsequent time points. We tested whether the intervention condition (i.e., GST versus GCBT as control condition) had a significant differential effect on SA at T2 (Path a, Figure 1), as well as on mediating variables at T1 (Path b, Figure 1), and whether mediating variables at T1 predicted SA at T2 (Path c, Figure 1). We tested mediation by using bootstrapping (*n* = 5000) to assess the indirect effect of GST versus GCBT on SA at T2 via mediating variables at T1 and to estimate 95% confidence intervals (CI) (Preacher and Hayes 2008). To establish mediation, the indirect path (Path b–c combined, Figure 1) must be significant (Zhao et al. 2010). As a check, we also assessed the indirect effect of GST versus GCBT on the mediator at T2 via SA symptoms at T1.

## 3 Results

### 3.1 Baseline Characteristics as Putative Moderating Variables

Baseline characteristics of the participants in GCBT and GST are described in Table 1 (for correlations, see Supplementary Table A3). No moderators of treatment response on the LSAS, AVPDSI and attrition were found. Both for the LSAS and the AVPDSI, none of the models with a three‐way interaction between condition, time and predictor showed a superior fit. For the Cox regression modelling time until dropout, none of the models with interactions between condition and each of the putative moderators was superior.

### 3.2 Differential Time Effects of Baseline Characteristics

#### 3.2.1 LSAS

An interaction between time and predictor at baseline was found for the LSAS with respect to the SMI‐AP scores and the frequency of psychological treatment in the 3 years preceding trial participation. The SMI‐AP was positively associated with the LSAS at all time points with a fixed effect estimate of 17.76. This estimate significantly decreased at T4 (17.76–8.27) and T5 (17.76–7.29). A similar pattern was found for patients with more than 20 therapy sessions prior to study entry. The estimate of the fixed effect (9.05) was significantly larger at T1, T2 and T3 (14.6, 13,47, 14.66, respectively) but not thereafter at T4 and T5 (4,61, 5,88, respectively) (see Supplementary Tables A4a–b for fixed effects).

#### 3.2.2 AVPDSI

The model with an interaction between predictor and time showed a better fit predicting treatment effects on the AVPDSI for the IDS, DERS, AAQ and RSES. The IDS and DERS had positive associations with the AVPDSI at baseline (0.39 and 0.16, respectively). At one‐year follow‐up, the parameter estimates were substantially reduced (IDS: −0.29, DERS: −0.15). Negative associations with the AVPDSI at baseline were found for the AAQ and RSES, which were largely reduced at one‐year follow‐up (−0.41 and 0.26, −0.59 and 0.61, respectively; see Supplementary Tables A5a–d for fixed effects parameters).

#### 3.2.3 Hazard of Attrition

No differential time effects of separate characteristics were found for the hazard of attrition.

### 3.3 Multivariate Analyses of Moderation of Baseline Characteristics

#### 3.3.1 LSAS

**FIGURE 2:**
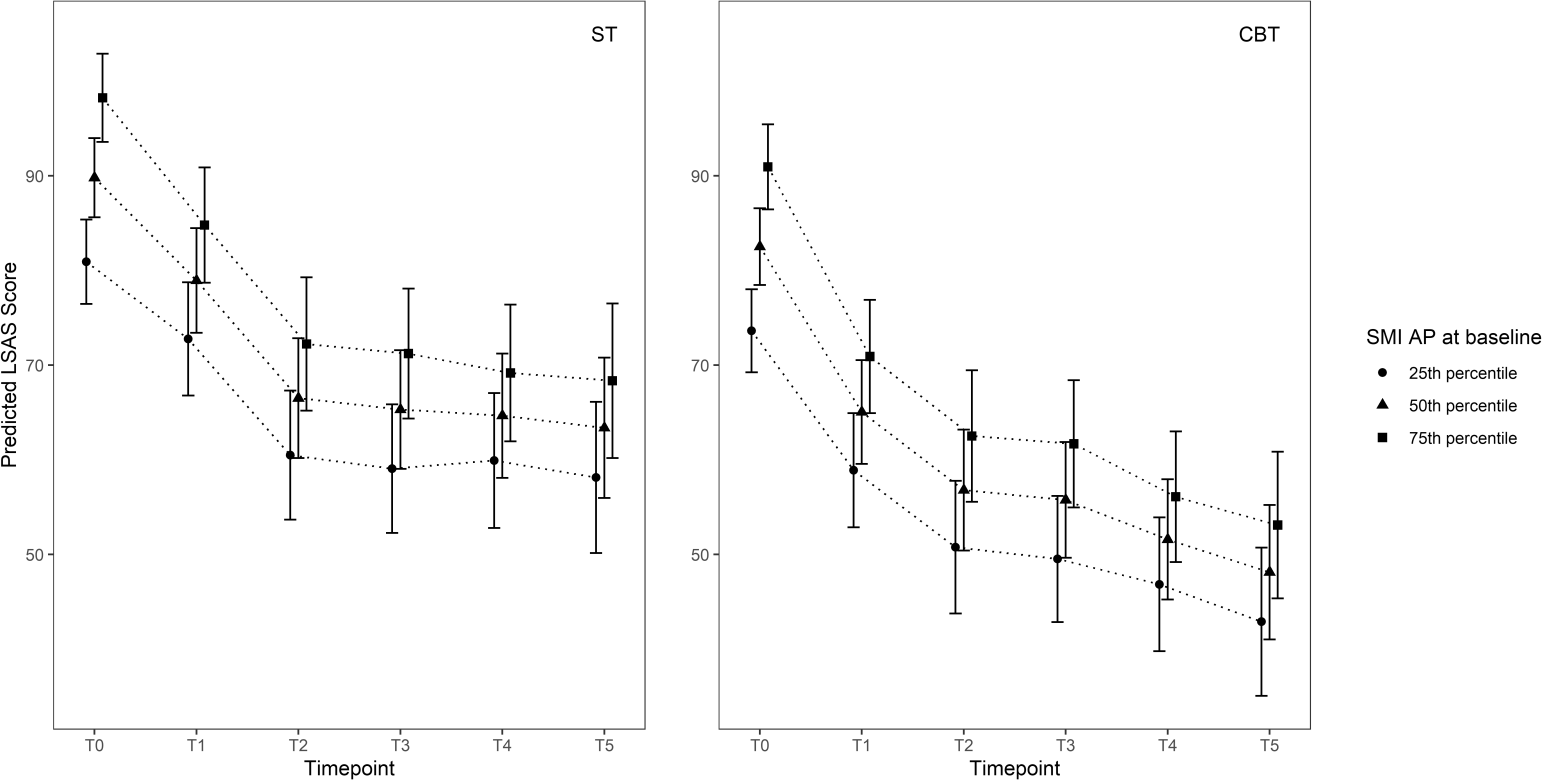
Multivariate multilevel analyses LSAS: Condition * time + SMI‐AP * time. Note: Figure 2 illustrates the differential time effects for patients at the 25th, 50th, and 75th percentiles of the Schema Mode Inventory‐Avoidant Protector (SMI‐AP). Predictor * time is an extension of the condition * time model; therefore, graphs are given for both conditions. However, condition * time * outcome was not significant.

Since no variables individually moderated the treatment effect on the LSAS, we examined possible combinations of the above‐mentioned time‐predictor interactions in a multivariate multilevel model. The superior model (i.e., with the lowest BIC) for the LSAS was a model including: condition * time + SMI‐AP * time (see Figure 2).

#### 3.3.2 AVPDSI

**FIGURE 3:**
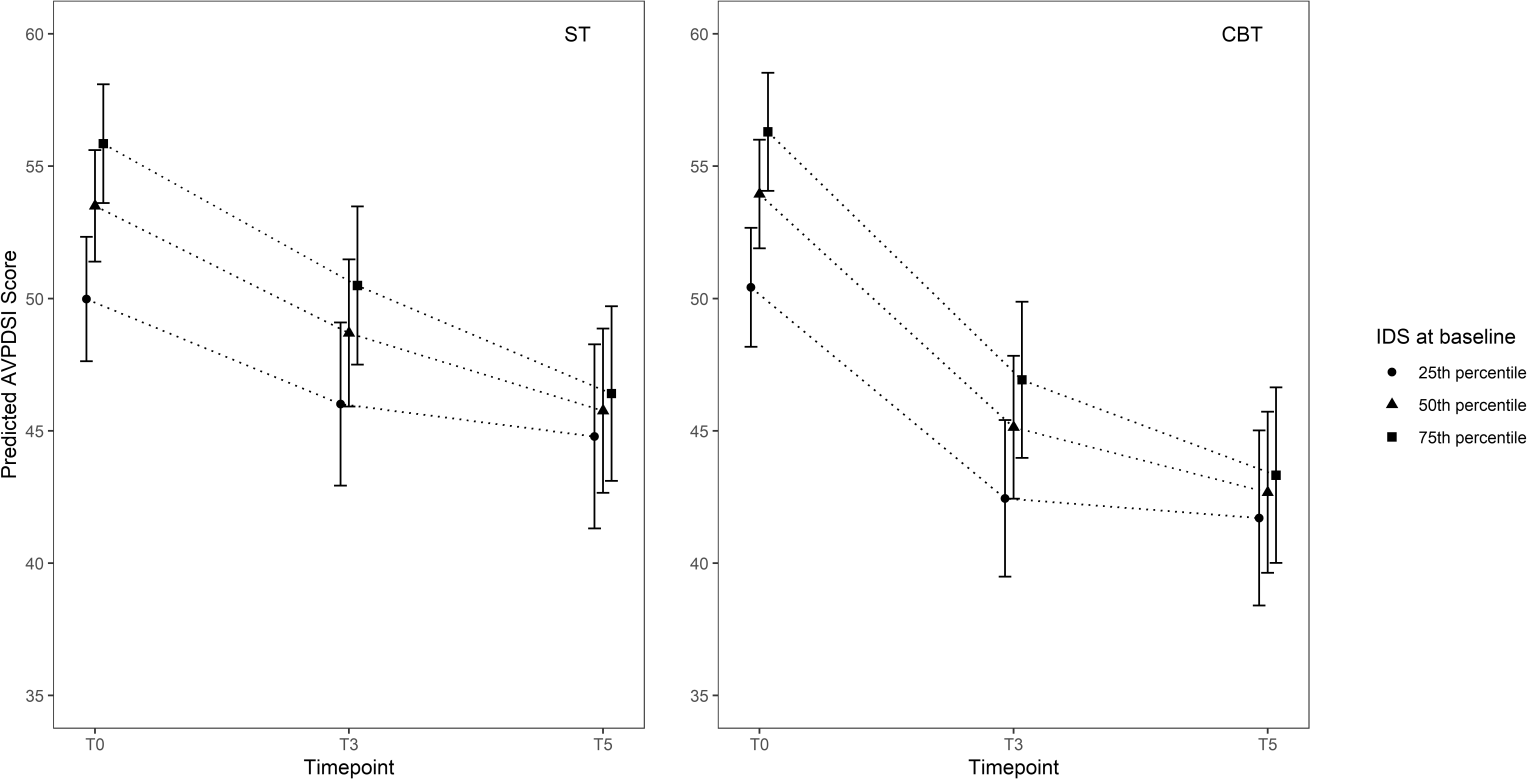
Multivariate multilevel analyses AVPDSI: Condition * time + IDS * time. Note: Figure 3 illustrates the differential time effects for patients on the Inventory of Depressive Symptomatology (IDS) at the 25th, 50th, and 75th percentiles. Predictor * time is an extension of the condition * time model; therefore, graphs are given for both conditions. However, condition * time * outcome was not significant.

All combinations of time‐predictor interactions for the IDS, AAQ, DERS, and RSES were examined in a multivariate multilevel model. None of the more complicated multivariate models outperformed a model with only one time * predictor term. The superior model (i.e., with the lowest BIC) for the AVPDSI was as follows: condition * time + IDS*time (see Figure 3).

#### 3.3.3 Hazard of Attrition

Combinations of the different baseline characteristics predicting hazard of attrition (see Supplementary Tables A6a–e) were examined in a multivariate model. Included were the IDS, physical neglect, emotional abuse and physical abuse (CTQ), and being married/cohabiting. The model with the lowest BIC contained main effects for emotional abuse (HR = 1.08) and being married/cohabiting (HR = 2.04).

In sum, no multivariate moderation of treatment response on the LSAS, AVPDSI and attrition was found.

### 3.4 Non‐Specific Predictors of Treatment Response

When added as a predictor to the time * condition model, several of the examined baseline variables were associated with constant better/worse outcomes on the LSAS and AVPDSI, as indicated by a significant main effect (see Supplementary Tables A7, A8a–l, A9a–j, Supplementary Figures A1–A2). Of the sociodemographic variables, education level and work status were associated with the LSAS, and being married/cohabiting with the AVPDSI. Of the clinical variables, the number of axis‐I disorders and avoidant and dependent traits were associated with both the LSAS and AVPDSI. Of the different measures, the IDS, AAQ, DERS and RSES were associated with the LSAS; the CTQ‐EN, CTQ‐EA and SMI‐AP were associated with the AVPDSI; and the SMI‐AV, SMI‐HA and SMI‐HC were associated with both the LSAS and AVPDSI. If a higher score on a variable represented dysfunction, the association was positive, and if a higher score was considered positive, the association was negative, e.g., more depressive symptoms (IDS) were associated with a higher LSAS score, and higher SE (RSES) was associated with a lower LSAS score.

### 3.5 Mediation Analyses

Multilevel analyses did not show differential treatment effects for GCBT and GST for the DERS, AAQ, RSES and SMI scores. For all putative mediating variables, we found main effects for time and no significant interactions between time and condition. Results of these multilevel analyses are summarised in Supplementary Tables A10 and A11.

The outcomes of the mediation analyses for the RI‐CLPM for each putative mediator are presented in Table 2. Each model showed satisfactory fit indices. No significant associations were found between the random intercepts of LSAS and each of the mediators. Regarding the autoregressive paths, all models showed statistically significant associations for the LSAS at T1 and T2, and for each of the putative mediators at T1 and T2. All models showed significant cross‐sectional associations at T2 between the LSAS and each of the putative mediators. Cross‐lagged effects for the LSAS at T2 were found with the RSES, SMI‐AV and SMI‐AP at T1. No association was found for the opposite relationships (i.e., LSAS at T1 with RSES, SMI‐AV, SMI‐AP at T2, respectively). None of the putative mediators were found to be actual mediators for the effect of condition on the LSAS. For completeness, we repeated the mediation analyses using a cross‐lagged panel model without random intercepts (see Supplementary Figure A3) and performed RI‐CLPM analyses of the per‐protocol sample of the RCT. These results (see Supplementary Tables A12, A13) also supported the absence of differential mediation in all investigated models.

**TABLE 2:** Outcomes of the random intercept cross‐lagged panel models examining the temporal and mediational relationships of the candidate mechanisms of change and social anxiety symptom severity.

	AAQ	DERS	RSES	SMI^a^	SMI‐HC	SMI‐HA	SMI‐AP
**Model fit**							
CFI	1.000	1.000	1.000	1.000	1.000	0.999	0.996
SRMR	0.003	0.007	0.015	0.003	0.012	0.017	0.014
**Association between random intercepts**							
RIanx with RIm	−0.248	0.585	−0.331	0.463	−0.380	−0.555	0.808
**Effect of condition**							
anx0 on cond	0.221	0.275	0.196	0.167	0.191	0.281	0.177
anx1 on cond	0.299	0.414	0.232	0.224	0.253	0.490	0.215
an2 on cond	0.002	0.031	−0.019	0.038	0.001	0.038	0.037
m0 on cond	−0.144	0.015	0.010	−0.015	−0.051	−0.016	−0.024
m1 on cond	−0.186	0.216	−0.096	0.145	−0.143	−0.251	0.048
m2 on cond	0.032	−0.040	0.062	−0.063	0.072	0.129	−0.030
**Cross‐sectional associations**							
anx0 with m0	−0.618	0.127	−0.422	0.363	−0.483	0.159	0.569
anx1 with m1	−0.389	0.127	−0.372	0.315	−0.350	0.131	0.535
anx2 with m2	−0.541 ***	0.551 ***	−0.621 ***	0.576***	−0.566 ***	−0.639 ***	0.702 ***
**Autoregressive paths**							
anx1 on anx0	0.102	−0.022	0.281	0.501	0.390	−0.247	0.539
anx2 on anx1	0.471 **	0.481 **	0.430 **	0.492**	0.573 **	0.442 **	0.542 **
m1 on m0	0.537	0.471	0.108	0.501	0.536	0.085	0.509
m2 on m1	0.552 **	0.481 **	0.485 *	0.599***	0.519 **	0.458 *	0.594 ***
**Cross‐lagged effects**							
anx1 on m0	−0.264	−0.022	−0.007	−0.086	−0.062	0.416	−0.086
anx2 on m1	−0.249	0.181	−0.351 **	0.308*	−0.138	−0.154	0.222 *
m1 on anx0	0.120	−0.411	−0.375	−0.056	−0.042	0.332	0.094
m2 on anx1	−0.155	0.162	−0.121	0.129	−0.215	−0.172	0.126
**Indirect mediation effects**							
cond to anx2 via m1	0.046 (−0.128;0.222)	0.039 (−0.102;0.180)	0.034 (−0.079;0.147)	0.045 (−0.101;0.190)	0.020 (−0.065;0.105)	0.039 (−0.053;0.130)	0.011 (−0.108;0.130)
cond to m2 via anx1	−0.046 (−255;0.162)	0.067 (−0.147;0.281)	−0.028 (−0.158;0.102)	0.029 (−0.083;0.140)	−0.054 (−0.241;0.132)	−0.084 (−0.252;0.084)	0.027 (−0.175;0.229)

Abbreviations: AAQ = Acceptance and Action Questionnaire‐II, anx = social anxiety symptom severity, AP = avoidant protector mode, CFI = comparative fit index, cond = condition, DERS = Difficulties in Emotion Regulation Scale, HA = healthy adult mode, HC = happy child mode, m = candidate mediator, RSES = Rosenberg Self‐Esteem Scale, SMI = Schema Mode Inventory, SRMR = standardised root mean squared residual.

^a^ Average SMI score.

* *p* < 0.05.

** *p* < 0.01.

*** *p* < 0.001.

## 4 Discussion

The current study aimed to explore differential moderators and mediators of treatment outcome in the context of an RCT comparing GCBT and GST for patients with SAD and AVPD. No moderators were identified, indicating that changes in SA symptoms, AVPD severity and attrition were not differentially related to the patient characteristics examined. In other words, none of the explored characteristics were related to response or attrition differences between GST and GCBT.

An interesting finding was that some patients who were more impaired at baseline, as shown by the association of certain characteristics with baseline severity levels of SA symptoms and AVPD manifestations, over time benefited relatively more from treatment than those who were less impaired. After a year, the impact of lower SE and ER difficulties on AVPD manifestations disappeared, while that of increased EA and depressive symptoms was greatly reduced. At the one‐year follow‐up, the significant impact of more extensive previous treatment for SA symptoms disappeared, and the impact of a strong AP mode was partly reduced. The aforementioned characteristics do not, or only minimally, impede achieving AVPD and SA outcomes comparable to those observed in less impaired patients. In addition, we also found predictor variables with a constant time effect, i.e., more dysfunction at baseline was associated with more SA symptoms (e.g., no work/study), more AVPD manifestations over time (e.g., high average mode score) and/or a higher hazard of attrition (e.g., being married/cohabiting) and vice versa. In conclusion, while some characteristics enabled patients at one‐year follow‐up to achieve comparable SAD and AVPD outcomes as less impaired patients, other characteristics may indicate that more impaired patients need more time or treatment to achieve similar outcomes as less impaired patients.

Furthermore, we examined candidate mediators with respect to SA symptoms during treatment. The formal mediation analyses showed that none of the indirect paths from treatment to SA at post‐treatment through the putative mediators at mid‐treatment were statistically significant. Changes in ER, EA, SE or SMs (HA, HC, AP, SMI‐average) did not mediate the effects of GST and GCBT on SA symptoms. This suggests a lack of evidence for differences between GCBT and GST in the underlying treatment processes related to the mediators under investigation.

Since RI‐CLPM separates within‐person variance from between‐person variance, it enables statements regarding within‐person processes, as these can represent causal effects over time. All the lagged relations pertain to within‐person fluctuations (Hamaker et al. 2015; Mulder and Hamaker 2021). At mid‐treatment, three putative mediators—SE, SMI‐AV and SMI‐AP—significantly predicted SA scores at post‐treatment, regardless of condition. Bidirectionality was ruled out. This suggests that aiming treatment at increasing SE, strengthening healthy modes while weakening dysfunctional modes (contributing to the SMI‐AV), with specific attention for reducing the AP, might positively impact SA symptoms at a later time.

Hofmann and Hayes (2019) distinguish therapeutic procedures from therapeutic processes. Therapeutic procedures are the techniques that a therapist utilises to achieve the client's treatment goal. Therapeutic processes are the underlying mechanisms that lead to the attainment of a desired goal. The prediction of later changes in SA in our SAD‐AVPD sample, irrespective of treatment, by SE, the SMI‐AV and the AP‐mode, may indicate more generic therapeutic processes for this population. Both GST and GCBT use different therapeutic procedures but may realise their effects on SA symptoms through similar underlying processes. In general, we consider them to be promising variables for future process studies in patients with SAD and AVPD.

Our findings on SMs are broadly in line with Yakin et al. (2020), who also found changes in modes to predict subsequent outcomes irrespective of treatment model. The prediction of later PD severity by HA and VC led them to conclude that these are central to the change process and appear to reflect common mechanisms of change. Although both studies differ in terms of outcome measures, time points of measurement, sample, treatments and statistical methods used, the similarity of the findings suggests that SMs may help us understand how treatment enables patients to improve on relevant outcomes.

In GCBT, SA was targeted by exposure to social situations and disconfirming dysfunctional beliefs. GST aimed to help patients to better meet their needs. The AP was the most prevalent mode. GST strives to reduce this coping mode directly, but also indirectly by addressing the modes that elicit the AP. Although different therapeutic procedures were employed, at mid‐treatment the AP was reduced in both conditions, which could explain why it was not a differential mediator. SMs could be crucial generic therapeutic processes for this severely avoidant population. Further research into SMs and their relationships may provide insight into processes affecting treatment outcomes.

The finding of mid‐treatment SE to predict SA symptoms at the end of treatment might be indicative of SE as a third potential process of interest. A recent review by Orth and Robins (2022) on the benefits of high (vs. low) SE for individuals and important life domains found that high (vs. low) SE has wide‐ranging positive consequences in different life domains, including mental health. Gathier et al. (2024) found SE, although cross‐sectionally, to mediate the relation between childhood trauma and anxiety and depression severity in a large adult sample (*n* = 1479), and they point out SE as a potentially relevant treatment target. Goldin et al. (2014) found that increased positive self‐views mediated the effect of CBT on social anxiety reduction. In sum, investigating how SE is modified by clinical interventions and its relation to treatment outcomes might facilitate optimisation of treatment outcomes. Since low SE is a pivotal diagnostic feature of AVPD (DSM‐IV/5) for patients with both SAD and AVPD, further studies on the role of SE might enhance the effectiveness of specific interventions.

Important strengths of this study were that it fulfilled multiple criteria for mechanism research (Lemmens et al. 2016), by using a RCT design that included a comparison group, a sufficient sample size (*n* > 40), examining multiple potential mediators, and the assessment of temporality. Second, mediators were measured by psychometrically valid instruments. Third, we used RI‐CLPM to investigate mediation, a specialised technique disentangling trait‐like and state‐like components of mechanisms of change. The latter reflects within‐client processes of change and is thereby suited to highlight active ingredients of successful treatment (Zilcha‐Mano 2021). We realised that our study examined a considerable number of moderating and mediating variables. However, given the scarcity of treatment studies on patients with SAD and comorbid AVPD (Lampe 2016, Simonsen et al. 2019; Weinbrecht et al. 2016), the resource‐intensive nature of RCTs (Griessbach et al. 2024), the need for RCTs meeting (as many of) the criteria for mechanism research (Lemmens et al. 2016) and the exploratory nature of our study, this approach seemed justifiable. Also, when selecting moderators and mediators, numerous variables can be considered (Hayes et al. 2022; Lutz et al. 2021). Furthermore, regarding ST, knowledge of the mechanisms of change and of which patients can benefit most from it is still in its infancy, justifying a more explorative approach (Fassbinder and Arntz 2021; Yakin et al. 2020).

Our study also has limitations. Since our goal was explorative, we did not control for the overall probability of a Type‐I error for multiple hypothesis tests. When selecting the best fitting parsimonious multivariate model we used the conservative BIC and did not interpret *p*‐values; however, the presence of any moderators in the final model might still be due to chance capitalisation. We did not experimentally manipulate putative mediators. Although influencing SMs was one of the goals of GST treatment, this is part of a complex therapeutic multi‐facetted process and as such cannot be seen as an isolated manipulation of individual SMs. Furthermore, putative moderators and mediators were all measured with self‐report questionnaires. In addition, the mediation analyses only included limited time points, with only the second of the three time points taking place during treatment. This was because the RCTs' main goal was to compare GCBT and GST, and maximising the response rate was already challenging in this avoidant sample, with the assessment battery being quite burdensome. Furthermore, our sample size may have been too small and may explain our null findings for differential mediators and moderators. The power problem of comparative outcome trials may be solved by meta‐analyses that include results of multiple trials. To improve and tailor psychological treatment many individual studies on moderators and mediators are needed (Cuijpers et al. 2019). Next, we did not include a non‐active control group. This would be necessary to investigate if treatment effects on social anxiety symptoms are mediated by SE, the SMI‐AV and the AP‐mode, irrespective of treatment modality. Finally, for each of the putative mediators only their unique influence was assessed. Psychotherapy is a multi‐dimensional phenomenon that might work through interplay of multiple mechanisms at several levels. As a result, it might be too complex to be explained in relatively simple causal models of psychological change (Lemmens et al. 2016).

### 4.1 Conclusions

First, the absence of moderators suggests that patient preference can be given a large role in the shared decision‐making process, as both GCBT and GST are effective treatments for comorbid SAD‐AVPD. Second, initial comorbid impairment by depressive symptoms, ER deficits, EA, and a lower quality of life is no reason to refrain from targeted treatment of AVPD. Third, some predictors were associated with consistently higher levels of SA symptoms and/or AVPD manifestations over time. Whether more treatment sessions or the addition of individual sessions to group treatment may be beneficial for more severely impaired patients requires future research. Finally, attention to improving SE, achieving a more functional mode constellation, and reducing the AP‐mode may help to realise later improvement, thereby paving the way for a life in which avoidance and social anxiety play an increasingly minor role. In the endeavour to unravel processes to make treatments more effective, further longitudinal studies applying more fine‐grained designs are highly needed.

## Author Contributions

**Astrid E. Baljé:** conceptualization, data curation, formal analysis, investigation, methodology, project administration, writing – original draft, writing – review and editing. **Anja Greeven:** conceptualization, data curation, funding acquisition, methodology, project administration, supervision, writing – review and editing. **Mathijs Deen:** formal analysis, methodology, writing – review and editing. **Anne E. van Giezen:** conceptualization, data curation, funding acquisition, methodology, project administration, supervision, writing – review and editing. **Arnoud Arntz:** conceptualization, data curation, formal analysis, methodology, supervision, writing – review and editing. **Philip Spinhoven:** conceptualization, data curation, formal analysis, funding acquisition, methodology, supervision, writing – review and editing.

## Disclosure

The study was approved by the relevant Institutional Review Boards (protocol number p12.165). Data were collected as part of a registered randomised controlled trial (NTR3921) and the study protocol and RCT results have been published.

## Ethics Statement

All participants provided online informed consent. We have complied with the ethical standards of the Declaration of Helsinki.

## Conflicts of Interest

The authors declare no conflicts of interest.

## Supporting information


**Data S1:** Supporting Information.

## Data Availability

The authors do not have permission to share data.
