# Supplementary material for: Exploring Moderators and Mediators of the Outcome of Group Cognitive Behavioural Therapy Compared With Group Schema Therapy for Social Anxiety Disorder and Comorbid Avoidant Personality Disorder
Source: Clin Psychol Psychother. 2025 Oct 13;32(5):e70148. doi: 10.1002/cpp.70148 (PMC12516937; doi:10.1002/cpp.70148)

Supplementary material to

**Exploring moderators and mediators of the outcome of Group Cognitive Behavioural Therapy compared With Group Schema Therapy for Social Anxiety Disorder and comorbid Avoidant Personality Disorder**

| **Supplementary material** | | | |
| --- | --- | --- | --- |
| Supplementary table A1 | | PCA one-component model: SMI modes, component loadings and communalities | 1 |
| Supplementary table A2 | | Main outcomes and candidate moderators and mediators: Cronbach alphas of measures/scales at different timepoints included in different analyses. | 2 |
| Supplementary table A3 | | Correlations between putative moderators and/or mediators | 3 |
| Supplementary Tables A4 | | Differential time effects of baseline characteristics for the LSAS | 4 |
|  | *Supplementary table A4a* | *LSAS, interaction between time and the avoidant protector mode (SMI-AP) at baseline: Anova and fixed effects* | *4* |
|  | *Supplementary table A4b* | *LSAS, interaction between time and number of psychological treatment sessions in the past 3 years at baseline: Anova & fixed effects* | *5* |
| Supplementary Tables A5 | | Differential time effects of baseline characteristics for the AVPDSI | 6 |
|  | *Supplementary table A5a* | *AVPDSI, interaction between time and IDS at baseline: Anova and fixed effects* | *6* |
|  | *Supplementary table A5b* | *AVPDSI, interaction between time and DERS at baseline: Anova and fixed effects* | *6* |
|  | *Supplementary table A5c* | *AVPDSI, interaction between time and AAQ at baseline: Anova and fixed effects* | *7* |
|  | *Supplementary table A5d* | *AVPDSI, interaction between time and RSES at baseline: Anova and fixed effects* | *7* |
| Supplementary Tables A6 | | Effects of baseline characteristics for the hazard of attrition | 8 |
|  | *Supplementary Table A6a* | *Inventory of depressive symptomatology: Estimated parameters for Cox regression regarding baseline scores* | *8* |
|  | *Supplementary Table A6b* | *CTQ physical neglect: Estimated parameters for Cox regression regarding baseline scores* | *8* |
|  | *Supplementary Table A6c* | *CTQ emotional abuse: Estimated parameters for Cox regression regarding baseline scores* | *8* |
|  | *Supplementary Table A6d* | *CTQ physical abuse: Estimated parameters for Cox regression regarding baseline scores* | *8* |
|  | *Supplementary Table A6e* | *Being married/cohabiting: Estimated parameters for Cox regression regarding baseline scores* | *8* |
| Supplementary Table A7 | | Model comparisons for the Liebowitz Social Anxiety Scale, the Avoidant personality disorder severity index and Treatment attrition | 9 |
| Supplementary Tables A8 | | LSAS, main effect of predictor | 10 |
|  | *Supplementary Table A8-a* | *LSAS, main effect of work and/or study* | *10* |
|  | *Supplementary Table A8-b* | *LSAS, main effect of education level* | *10* |
|  | *Supplementary Table A8-c* | *LSAS, main effect of number of symptom disorders* | *11* |
|  | *Supplementary Table A8-d* | *LSAS, main effect of traits of avoidant personality disorder* | *11* |
|  | *Supplementary Table A8-e* | *LSAS, main effect of traits of dependent personality disorder* | *12* |
|  | *Supplementary Table A8-f* | *LSAS, main effect of Inventory of depressive symptomatology* | *12* |
|  | *Supplementary Table A8-g* | *LSAS, main effect of Acceptance and Action Questionnaire* | *13* |
|  | *Supplementary Table A8-h* | *LSAS, main effect of Difficulties in Emotion Regulation Scale* | *13* |
|  | *Supplementary Table A8-i* | *LSAS, main effect of Rosenberg Self-Esteem Scale* | *14* |
|  | *Supplementary Table A8-j* | *LSAS, main effect of Schema mode inventory - average score* | *14* |
|  | *Supplementary Table A8-k* | *LSAS, main effect of Schema mode inventory - Healthy Adult* | *15* |
|  | *Supplementary Table A8-l* | *LSAS, main effect of Schema mode inventory - Happy Child* | *15* |
| Supplementary Tables A9 | | AVPDSI, main effect of predictor | 16 |
|  | *Supplementary Table A9-a* | *AVPDSI, Main effect of being married/cohabiting* | *16* |
|  | *Supplementary Table A9-b* | *AVPDSI, Main effect of number of symptom disorders* | *16* |
|  | *Supplementary Table A9-c* | *AVPDSI, Main effect of traits of avoidant personality disorder* | *17* |
|  | *Supplementary Table A9-d* | *AVPDSI, Main effect of traits of dependent personality disorder* | *17* |
|  | *Supplementary Table A9-e* | *AVPDSI, Main effect of Childhood Trauma Questionnaire - emotional neglect* | *18* |
|  | *Supplementary Table A9-f* | *AVPDSI, Main effect of Childhood Trauma Questionnaire - emotional abuse* | *18* |
|  | *Supplementary Table A9-g* | *AVPDSI, Main effect of Schema mode inventory – average score* | *19* |
|  | *Supplementary Table A9-h* | *AVPDSI, Main effect of Schema mode inventory – avoidant protector* | *19* |
|  | *Supplementary Table A9-i* | *AVPDSI, Main effect of Schema mode inventory – healthy adult* | *20* |
|  | *Supplementary Table A9-j* | *AVPDSI, Main effect of Schema mode inventory – happy child* | *20* |
| Supplementary Figure A1 | | Multivariate multilevel analyses LSAS: Condition * time + IDS | 21 |
| Supplementary Figure A2 | | Multivariate multilevel analyses AVPDSI: Condition * time + SMI-HC | 21 |
| Supplementary Table A10 | | Anova, effect of treatment on putative mediating variables: DERS, RSES, AAQ, SMI and SMI scales | 22 |
| Supplementary Table A11 | | Estimated means and within-group effect sizes AAQ, DERS, RSES and SMI per condition, intention-to-treat sample. | 23 |
| Supplementary Table A12 | | Outcomes of cross-lagged panel models examining the temporal and mediational relationships of candidate mechanisms of change and social anxiety symptom severity. | 24 |
| Supplementary Table A13 | | Per-protocol sample: Outcomes of random- intercept cross-lagged panel models examining the temporal and mediational relationships of candidate mechanisms of change and social anxiety symptom severity. | 25 |
| Supplementary Figure A3 | | Cross-lagged panel models examining the temporal and mediational relationships of candidate mechanisms of change and social anxiety symptom severity. | 26 |

**Supplementary table A1**

PCA one-component model: SMI modes, component loadings and communalities

| Schema mode | M | SD | alpha | No. of items | Communalities^a^ | Loadings^b^ |
| --- | --- | --- | --- | --- | --- | --- |
| Lonely child | 3,47 | 0.87 | .89 | 11 | .62 | .79 |
| Abandoned and abused child | 3.71 | 0.83 | .87 | 12 | .76 | .87 |
| Angry child | 2.57 | 0.84 | .87 | 11 | .45 | .67 |
| Enraged child | 1.68 | 0.62 | .79 | 7 | .26 | .51 |
| Impulsive child | 2.41 | 0.73 | .81 | 8 | .28 | .53 |
| Undisciplined child | 2.90 | 0.71 | .68 | 7 | .55 | .74 |
| Dependent child | 2.97 | 0.82 | .84 | 10 | .53 | .73 |
| Compliant surrender | 3.82 | 0.93 | .87 | 9 | .44 | .66 |
| Detached protector | 3.16 | 0.80 | .87 | 13 | .57 | .75 |
| Detached self-soother | 3.20 | 0.72 | .67 | 9 | .40 | .63 |
| Avoidant protector | 4.27 | 0.78 | .85 | 10 | .45 | .67 |
| Self aggrandizer | 2.37 | 0.69 | .77 | 10 | .19 | .44 |
| Perfectionistic overcontroller | 3.55 | 0.73 | .74 | 10 | .28 | .53 |
| Suspicious overcontroller | 3.42 | 0.96 | .88 | 9 | .45 | .67 |
| Attention and approval seeker | 1.57 | 0.56 | .74 | 6 | .00 | .06 |
| Punitive parent | 3.00 | 0.83 | .87 | 11 | .67 | .82 |
| Demanding parent | 3.66 | 0.85 | .82 | 10 | .19 | .44 |
| Healthy adult | 4.04 | 0.65 | .78 | 11 | .53 | .73 |
| Happy child | 4.22 | 0.65 | .81 | 10 | .40 | .64 |

Note: ^a^ Communalities indicate the amount of variance in each variable that is accounted for; ^b^ Component loadings are the correlations between the variable and the component. No = number. Explanatory note: The SMI-2 measures 18 SMs. We added the HC mode from the SMI-1 (SMI; Lobbestael et al., 2010) because of its putative therapeutic relevance in the ST treatment for patients with AVPD. We reduced the data by first performing a principal component analysis (PCA) with varimax rotation in SPSS on the SMI subscale scores at baseline. This was done to reduce the number of moderator and mediator analyses examining differential effects on study outcomes with respect to the SMI. Scores of the HA and HC modes were reversed. Based on a combination of the scree plot, correlational data, and interpretability, this PCA revealed a one-component model as a suitable and interpretable solution. The variance accounted for was 42.3 %, and all but one component loadings were higher than .4 (see Supplementary Table A1). Results of PCAs extracting a higher number of components yielded uninterpretable results. Therefore, a composite measure was used for all SMs by computing an average score of the means on all subscales, with a higher score representing a less favorable score, referred to as SMI-AV.

**Supplementary table A2**

Main outcomes and candidate moderators and mediators: Cronbach alphas of measures/scales at different timepoints included in different analyses.

|  | | | **wave A** | **wave B** | **wave C** | **wave D** | **wave E** | **wave F** |
| --- | --- | --- | --- | --- | --- | --- | --- | --- |
| **Main outcomes** | | |  |  |  |  |  |  |
|  | LSAS | | 0.94 | 0.95 | 0.97 | 0.97 | 0.97 | 0.98 |
|  | AVDPSI | | 0.91 | - | - | 0.95 | - | 0.95 |
| **Candidate mediators/ moderators** | | |  |  |  |  |  |  |
|  | RSES | | 0.82 | 0.89 | 0.91 | 0.91 | 0.90 | 0.93 |
|  | DERS^a^ | | 0.94 | 0.93 | 0.95 | 0.96 | 0.96 | 0.96 |
|  | AAQ | | 0.83 | 0.87 | 0.90 | 0.92 | 0.91 | 0.93 |
|  | SMI all items | | 0.97 | 0.98 | 0.99 | 0.99 | 0.99 | 0.99 |
|  | SMI-HA | | 0.78 | 0.82 | 0.88 | 0.91 | 0.91 | 0.90 |
|  | SMI-HC | | 0.81 | 0.83 | 0.88 | 0.92 | 0.88 | 0.92 |
|  | SMI-AP | | 0.85 | 0.87 | 0.90 | 0.91 | 0.92 | 0.92 |
| **Candidate moderators** | | |  |  |  |  |  |  |
|  | IDS | | 0.87 | - | - | - | - | - |
|  | CTQ | |  | - | - | - | - | - |
|  |  | emotional neglect | 0.91 | - | - | - | - | - |
|  |  | physical neglect | 0.72 | - | - | - | - | - |
|  |  | emotional abuse | 0.89 | - | - | - | - | - |
|  |  | physical abuse | 0.79 | - | - | - | - | - |
|  |  | sexual abuse | 0.89 | - | - | - | - | - |

LSAS = Liebowitz Social Anxiety Scale, AVPDSI = Avoidant Personality Disorder Severity Index, RSES = Rosenberg Self Esteem Scale, DERS = Difficulties in Emotion Regulation Scale; AAQ = Acceptance and Action Questionnaire, SMI = schema mode inventory, SMI-HA = SMI healthy adult mode, SMI-HC = SMI happy child mode, SMI-AP = SMI avoidant protector mode. IDS = Inventory of Depressive Symptomatology, - = not applicable; ^a^ DERS awareness items are excluded (Hallion et.al, 2018).

**Supplementary Table A3** Correlations between putative moderators and/or mediator

|  | Education  level | Work or  study | Married/  cohabiting | Medication | PT last  3 years | Depressive  disorder | No. axis I  disorders | No. AVPD  disorders | No. DPD  traits | No. OCPD  traits | No. BPD  traits | CTQ-EN | CTQ-N | CTQ-EA | CTQ-PA | CTQSA | IDS | AAQ | DERS | RSES | SMI-AV | SMI-AP | SMI-HA | SMI-HC |
| --- | --- | --- | --- | --- | --- | --- | --- | --- | --- | --- | --- | --- | --- | --- | --- | --- | --- | --- | --- | --- | --- | --- | --- | --- |
| Education level | -- |  |  |  |  |  |  |  |  |  |  |  |  |  |  |  |  |  |  |  |  |  |  |  |
| Work or study Y/N | -.26** | -- |  |  |  |  |  |  |  |  |  |  |  |  |  |  |  |  |  |  |  |  |  |  |
| Married/cohabiting Y/N | .05 | -.10 | -- |  |  |  |  |  |  |  |  |  |  |  |  |  |  |  |  |  |  |  |  |  |
| Medication Y/N | .03 | .16 | -.03 | -- |  |  |  |  |  |  |  |  |  |  |  |  |  |  |  |  |  |  |  |  |
| Psychological treatment  last three years | -,04 | -.13 | -.03 | -.30** | -- |  |  |  |  |  |  |  |  |  |  |  |  |  |  |  |  |  |  |  |
| Depressive disorder Y/N | .04 | -.10 | .08 | -.09 | .01 | -- |  |  |  |  |  |  |  |  |  |  |  |  |  |  |  |  |  |  |
| Number axis I disorders | .01 | -.06 | .01 | -.12 | .09 | .50** | -- |  |  |  |  |  |  |  |  |  |  |  |  |  |  |  |  |  |
| Number AVPD traits | -.06 | -.15 | -.03 | -.10 | .13 | .02 | .03 | -- |  |  |  |  |  |  |  |  |  |  |  |  |  |  |  |  |
| Number DPD traits | -.09 | -.04 | .13 | .03 | -.08 | .17* | .10 | .13 | -- |  |  |  |  |  |  |  |  |  |  |  |  |  |  |  |
| Number OCD traits | .03 | .04 | .12 | -.13 | -.04 | .14 | .15 | .08 | .02 | -- |  |  |  |  |  |  |  |  |  |  |  |  |  |  |
| Number BPD traits | -.09 | .04 | .14 | .01 | .03 | .15 | .13 | .01 | .26** | .13 | -- |  |  |  |  |  |  |  |  |  |  |  |  |  |
| CTQ-EN | -.04 | .18* | .06 | .09 | -.09 | -.03 | .03 | .20* | .00 | .07 | .07 | -- |  |  |  |  |  |  |  |  |  |  |  |  |
| CTQ-PN | -.11 | .11 | .18* | .09 | -.11 | .03 | .06 | .13 | .15 | .14 | .11 | .73** | -- |  |  |  |  |  |  |  |  |  |  |  |
| CTQ-EA | -.13 | .06 | .04 | .08 | -.07 | .00 | .09 | .19* | .13 | .11 | .14 | .73** | .66** | -- |  |  |  |  |  |  |  |  |  |  |
| CTQ-PA | -.09 | .03 | .04 | .01 | -.12 | .00 | -.03 | .16 | -.04 | .24** | .02 | .47** | .45** | .57** | -- |  |  |  |  |  |  |  |  |  |
| CTQ-SA | .00 | -.05 | .07 | -.07 | -.02 | .05 | .07 | .13 | -.06 | .20* | .14 | .23** | .32** | .38** | .43** | -- |  |  |  |  |  |  |  |  |
| IDS | -.07 | -.21* | .09 | -.21** | .03 | .36** | .33** | .28** | .34** | .05 | .23** | .16* | .21** | .26** | .00 | .10 | -- |  |  |  |  |  |  |  |
| AAQ | -.01 | -.13 | -.03 | -.15 | .04 | .27** | .24** | .35** | .25** | .03 | .10 | .12 | .11 | .14 | -.02 | .13 | .58** | -- |  |  |  |  |  |  |
| DERS^a^ | .00 | -.08 | .13 | -.20* | .02 | .26** | .31** | .21* | .22** | .03 | .19* | .04 | .09 | .05 | -.09 | .06 | .59** | .71** | -- |  |  |  |  |  |
| RSES | -.06 | .11 | .01 | .04 | -.05 | -015 | -.16* | -.32** | -.21* | .07 | -.06 | -.18* | -.10 | -.19* | .06 | -.01 | -.48** | -.53** | -.44** | -- |  |  |  |  |
| SMI-Average | -.01 | .01 | .06 | -.10 | -.07 | .25** | .26** | .33** | .30** | .00 | .28** | .20* | .19* | .20* | .01 | .12 | .68** | .72** | .74** | -.54** | -- |  |  |  |
| SMI-AP | -.10 | -.17* | -.02 | -.02 | -.01 | .16* | .12 | .39** | .22** | -.03 | .15 | -.01 | .01 | .04 | -.06 | .10 | .52** | .52** | .46** | -.49** | .64** | -- |  |  |
| SMI-HA | -.06 | .06 | .03 | .10 | -.08 | -.21** | -.18* | -.26** | -.27** | .00 | -.08 | -.23** | -.20* | -.11 | .06 | -.05 | -.51** | -.63** | -.55** | .61** | -.69** | -.53** | -- |  |
| SMI-HC | -.01 | .04 | .14 | .01 | -.07 | -.10 | -.03 | -.34** | -.11 | .16* | -.03 | -.25** | -.17* | -.17* | .06 | -.06 | -.45** | -.52** | -.35** | -.50** | -.59** | -.51** | .70** | -- |

Abrreviations: Y/N = yes/no, CTQ = childhood trauma questionnaire, EN = emotional neglect, PN = physical neglect, EA = emotional abuse, PA = physical abuse, SA = sexual abuse, IDS = Inventory of Depressive Symptomatology, AAQ = Acceptance and Action Questionnaire, DERS = Difficulties in Emotion Regulation Scale, RSES = Rosenberg Self Esteem Scale, SMI = schema mode inventory, AV= average, SMI-AP = SMI avoidant protector, SMI-HA = SMI healthy adult, SMI-HC = SMI happy child, No.= number, -- = not applicable; ^a^ DERS awareness items are excluded (Hallion et.al, 2018).

**Supplementary Tables A4**

Differential time effects of baseline characteristics for the LSAS

**Supplementary table A4a**

LSAS, interaction between time and the avoidant protector mode (SMI-AP) at baseline: Anova and fixed effects

| **Anova** | | | **Num DF** | **F** | **p** |  | |  |
| --- | --- | --- | --- | --- | --- | --- | --- | --- |
| Intercept | | | 1 | 3272.47 | <.01 |  | |  |
| Condition | | | 1 | 7.02 | 0.01 |  | |  |
| Time | | | 5 | 45.34 | <.01 |  | |  |
| SMI-AP^a^ | | | 1 | 85.69 | <.01 |  | |  |
| Condition * time | | | 5 | 1.87 | 0.10 |  | |  |
| Time * SMI-AP | | | 5 | 2.75 | 0.02 |  | |  |
| **Fixed effects** | | | **B** | **SE** | **t** | | **df** | **p** |
| Intercept  Condition ST^a^  Time 1  Time 2  Time 3  Time 4  Time 5  SMI-AP  Time effect for condition ST | | | 82.37 | 2.06 | 39.97 | | 737 | <.01 |
|  |  |  | 7.29 | 2.96 | 2.46 | | 737 | 0.01 |
|  |  |  | -17.42 | 1.95 | -8.93 | | 737 | <.01 |
|  |  |  | -25.68 | 2.76 | -9.31 | | 737 | <.01 |
|  |  |  | -26.72 | 2.88 | -9.28 | | 737 | <.01 |
|  |  |  | -30.87 | 3.19 | -9.67 | | 737 | <.01 |
|  |  |  | -34.33 | 3.68 | -9.32 | | 737 | <.01 |
|  |  |  | **17.76** | 1.89 | 9.38 | | 737 | **<.01** |
|  |  |  |  |  |  | |  |  |
|  | Time 1  Time 2  Time 3  Time 4  Time 5 | | 6.61 | 2.74 | 2.41 | | 737 | 0.02 |
|  |  |  | 2.44 | 3.90 | 0.63 | | 737 | 0.53 |
|  |  |  | 2.26 | 4.19 | 0.54 | | 737 | 0.59 |
|  |  |  | 5.79 | 4.65 | 1.25 | | 737 | 0.21 |
|  |  |  | 7.96 | 5.37 | 1.48 | | 737 | 0.14 |
| Time effect for SMI AP | | |  |  |  | |  |  |
|  | | Time 1  Time 2  Time 3  Time 4  Time 5 | **-5.44** | 1.77 | -3.07 | | 737 | **<.01** |
|  |  |  | **-5.72** | 2.50 | -2.29 | | 737 | **0.02** |
|  |  |  | **-5.29** | 2.64 | -2.01 | | 737 | **0.05** |
|  |  |  | **-8.27** | 3.01 | -2.75 | | 737 | **0.01** |
|  |  |  | **-7.29** | 3.43 | -2.13 | | 737 | **0.03** |

Note. Cognitive Behavioral Therapy (CBT) was reference category. ^a^ SMI-AP was mean centered. SMI-AP = Schema mode inventory - Avoidant Protector; ST = Schema Therapy.

**Supplementary table A4b**

LSAS, interaction between time and number of psychological treatment sessions in the past 3 years at baseline: Anova & fixed effects

| **Anova** | **Num DF** |  | **F** | **p** |  | |  |
| --- | --- | --- | --- | --- | --- | --- | --- |
| Intercept | 1 |  | 2089.33 | <.01 |  | |  |
| Condition | 1 |  | 8.82 | <.01 |  | |  |
| Time | 5 |  | 39.08 | <.01 |  | |  |
| Number of sessions | 4 |  | 4.32 | <.01 |  | |  |
| Condition * time | 5 |  | 1.67 | 0.14 |  | |  |
| Time * number of sessions | 20 |  | 1.86 | 0.01 |  | |  |
| **Fixed effects** | **B** |  | **SE** | **t** | **df** | **p** | |
| Intercept | 78.15 |  | 3.71 | 21.05 | 719 | <.01 | |
| Condition ST | 6.98 |  | 3.71 | 1.88 | 719 | 0.06 | |
| Time 1 | -25.53 |  | 2.84 | -9.00 | 719 | <.01 | |
| Time 2 | -32.52 |  | 4.12 | -7.90 | 719 | <.01 | |
| Time 3 | -34.62 |  | 4.47 | -7.74 | 719 | <.01 | |
| Time 4 | -31.98 |  | 5.00 | -6.39 | 719 | <.01 | |
| Time 5 | -35.78 |  | 5.90 | -6.06 | 719 | <.01 | |
| Number of sessions 1 - 5 | -3.15 |  | 8.02 | -0.39 | 719 | 0.70 | |
| Number of sessions 6 - 10 | -0.08 |  | 7.03 | -0.01 | 719 | 0.99 | |
| Number of sessions 11 - 20 | 0.31 |  | 5.81 | 0.05 | 719 | 0.96 | |
| Number of sessions > 20 | **9.05** |  | 4.49 | 2.02 | 719 | **0.04** | |
| Condition ST * time 1 | 5.36 |  | 2.77 | 1.93 | 719 | 0.05 | |
| Condition ST * time 2 | 1.65 |  | 4.05 | 0.41 | 719 | 0.69 | |
| Condition ST * time 3 | 1.79 |  | 4.38 | 0.41 | 719 | 0.68 | |
| Condition ST * time 4 | 6.79 |  | 4.91 | 1.38 | 719 | 0.17 | |
| Condition ST * time 5 | 8.56 |  | 5.67 | 1.51 | 719 | 0.13 | |
| Time 1 * Number of sessions 1 - 5 | 3.52 |  | 5.95 | 0.59 | 719 | 0.55 | |
| Time 2 * Number of sessions 1 - 5 | 6.65 |  | 8.85 | 0.75 | 719 | 0.45 | |
| Time 3 * Number of sessions 1 - 5 | 6.90 |  | 9.83 | 0.70 | 719 | 0.48 | |
| Time 4 * Number of sessions 1 - 5 | 7.61 |  | 10.68 | 0.71 | 719 | 0.48 | |
| Time 5 * Number of sessions 1 - 5 | 13.35 |  | 12.30 | 1.09 | 719 | 0.28 | |
| Time 1 * Number of sessions 6 - 10 | 8.41 |  | 5.37 | 1.57 | 719 | 0.12 | |
| Time 2: Number of sessions 6 - 10 | 1.69 |  | 7.57 | 0.22 | 719 | 0.82 | |
| Time 3: Number of sessions 6 - 10 | 0.07 |  | 8.53 | 0.01 | 719 | 0.99 | |
| Time 4: Number of sessions 6 - 10 | -15.54 |  | 9.48 | -1.64 | 719 | 0.10 | |
| Time 5: Number of sessions 6 - 10 | -14.49 |  | 11.03 | -1.31 | 719 | 0.19 | |
| Time 1: Number of sessions 11 - 20 | 13.83 |  | 4.35 | 3.18 | 719 | <.01 | |
| Time 2: Number of sessions 11 - 20 | 10.83 |  | 6.36 | 1.70 | 719 | 0.09 | |
| Time 3: Number of sessions 11 - 20 | 13.68 |  | 6.74 | 2.03 | 719 | 0.04 | |
| Time 4: Number of sessions 11 - 20 | 1.84 |  | 7.71 | 0.24 | 719 | 0.81 | |
| Time 5: Number of sessions 11 - 20 | 0.16 |  | 8.82 | 0.02 | 719 | 0.99 | |
| Time 1: Number of sessions > 20 | 14.59 |  | 3.37 | 4.33 | 719 | <.01 | |
| Time 2: Number of sessions > 20 | 13.47 |  | 4.92 | 2.74 | 719 | 0.01 | |
| Time 3: Number of sessions > 20 | 14.66 |  | 5.36 | 2.74 | 719 | 0.01 | |
| Time 4: Number of sessions > 20 | 4.61 |  | 5.97 | 0.77 | 719 | 0.44 | |
| Time 5: Number of sessions > 20 | 5.88 |  | 6.98 | 0.84 | 719 | 0.40 | |

Note. Cognitive Behavioral Therapy (CBT) was reference category. ST = Schema Therapy.

**Supplementary Tables A5**

Differential time effects of baseline characteristics for the AVPDSI

**Supplementary Table A5a**

AVPDSI, interaction between time and IDS at baseline: Anova and fixed effects

| **Anova** | **Num DF** | **DenDF** | **F** | **p** |  |
| --- | --- | --- | --- | --- | --- |
| Intercept | 1 | 206 | 5701.89 | <.01 |  |
| Condition | 1 | 143 | 0.40 | 0.53 |  |
| Time | 2 | 206 | 49.95 | <.01 |  |
| IDS^a^ | 1 | 143 | 44.50 | <.01 |  |
| Condition * time | 2 | 206 | 2.58 | 0.08 |  |
| Time * IDS | 2 | 206 | 7.22 | **<.01** |  |
| **Fixed effects** | **AVPDSI** | **SE** | **t** | **df** | **p** |
| Intercept | 53.56 | 1.00 | 53.49 | 206 | <.01 |
| Condition ST | -0.37 | 1.44 | -0.26 | 143 | 0.80 |
| Time 1 | -8.71 | 1.25 | -6.99 | 206 | <.01 |
| Time 2 | -10.97 | 1.36 | -8.08 | 206 | <.01 |
| IDS | **0.39** | 0.06 | 6.62 | 143 | **<.01** |
| Condition ST * time 1 | 4.15 | 1.79 | 2.31 | 206 | 0.02 |
| Condition ST * time 2 | 2.91 | 1.95 | 1.49 | 206 | 0.14 |
| Time1 * IDS | -0.10 | 0.08 | -1.27 | 206 | 0.21 |
| Time 2 * IDS | **-0.29** | 0.08 | -3.55 | 206 | **<.01** |

Note. Cognitive Behavioral Therapy (CBT) was reference category. ^a^ IDS was mean centered, IDS = Inventory of Depressive Symptomatology, ST = Schema Therapy.

**Supplementary Table A5b**

AVPDSI, interaction between time and DERS at baseline: Anova and fixed effects

| **Anova** | **Num DF** | **DenDF** | **F** | **p** |  |
| --- | --- | --- | --- | --- | --- |
| Intercept | 1 | 206 | 4834.09 | <.01 |  |
| Condition | 1 | 143 | 0.32 | 0.57 |  |
| Time | 2 | 206 | 49.27 | <.01 |  |
| DERS^ab^ | 1 | 143 | 15.77 | <.01 |  |
| Condition * time | 2 | 206 | 2.46 | 0.09 |  |
| Time * DERS | 2 | 206 | 6.34 | <.01 |  |
| **Fixed effects** | **AVPDSI** | **SE** | **t** | **df** | **p** |
| Intercept | 53.47 | 1.07 | 49.78 | 206 | <.01 |
| Condition ST | -0.03 | 1.54 | -0.02 | 143 | 0.98 |
| Time 1 | -8.70 | 1.24 | -7.02 | 206 | <.01 |
| Time 2 | -10.85 | 1.37 | -7.93 | 206 | <.01 |
| DERS | **0.16** | 0.04 | 4.39 | 143 | **<.01** |
| Condition ST * time 1 | 4.05 | 1.78 | 2.27 | 206 | 0.02 |
| Condition ST * time 2 | 2.80 | 1.96 | 1.43 | 206 | 0.15 |
| Time 1 * DERS | -0.08 | 0.04 | -1.89 | 206 | 0.06 |
| Time 2 * DERS | **-0.15** | 0.04 | -3.52 | 206 | **<.01** |

Note. Cognitive Behavioral Therapy (CBT) was reference category. ^a^ DERS was mean centered,

ST = Schema Therapy, DERS = Difficulties in Emotion Regulation Scale, ^a^ DERS awareness items are excluded (Hallion et.al, 2018).

**Supplementary Table A5c**

AVPDSI, interaction between time and AAQ at baseline: Anova and fixed effects

| **Anova** | **Num DF** | **DenDF** | **F** | **p** |  |
| --- | --- | --- | --- | --- | --- |
| Intercept | 1 | 206 | 4979.29 | <.01 |  |
| Condition | 1 | 143 | 0.36 | 0.55 |  |
| Time | 2 | 206 | 46.79 | <.01 |  |
| AAQ^a^ | 1 | 143 | 20.80 | <.01 |  |
| Condition * time | 2 | 206 | 2.48 | 0.09 |  |
| Time * AAQ | 2 | 206 | 3.06 | 0.05 |  |
| **Fixed effects** | **AVPDSI** | **SE** | **t** | **df** | **p** |
| Intercept | 53.88 | 1.07 | 50.59 | 206 | <.01 |
| Condition ST | -0.85 | 1.53 | -0.55 | 143 | 0.58 |
| Time 1 | -8.81 | 1.25 | -7.06 | 206 | <.01 |
| Time 2 | -11.13 | 1.41 | -7.89 | 206 | <.01 |
| AAQ | **-0.41** | 0.08 | -4.83 | 143 | **<.01** |
| Condition ST * time 1 | 4.28 | 1.80 | 2.37 | 206 | 0.02 |
| Condition ST * time 2 | 3.32 | 2.03 | 1.63 | 206 | 0.10 |
| Time 1 * AAQ | 0.13 | 0.10 | 1.37 | 206 | 0.17 |
| Time 2 * AAQ | **0.26** | 0.11 | 2.46 | 206 | **0.02** |

Note. Cognitive Behavioral Therapy (CBT) was reference category. ^a^ AAQ was mean centered,

AAQ = Acceptance and Action Questionnaire, ST = Schema Therapy.

**Supplementary Table A5d**

AVPDSI, interaction between time and RSES at baseline: Anova and fixed effects

| **Anova** | **numDF** | **denDF** | **F** | **p** |  |
| --- | --- | --- | --- | --- | --- |
| Intercept | 1 | 206 | 4605.70 | <0.01 |  |
| Condition | 1 | 143 | 0.35 | 0.56 |  |
| Time | 2 | 206 | 47.53 | <0.01 |  |
| RSES^a^ | 1 | 143 | 8.49 | <0.01 |  |
| Condition * time | 2 | 206 | 2.48 | 0.09 |  |
| Time * RSES | 2 | 206 | 4.31 | 0.02 |  |
| **Fixed effects** | **AVPDSI** | **SE** | **t** | **df** | **p** |
| Intercept | 53.52 | 1.10 | 48.66 | 206 | <0.01 |
| Condition ST | -0.05 | 1.58 | -0.03 | 143 | 0.98 |
| Time1 | -8.68 | 1.24 | -7.00 | 206 | <0.01 |
| Time2 | -10.87 | 1.39 | -7.81 | 206 | <0.01 |
| RSES | **-0.59** | 0.17 | -3.44 | 143 | **<0.01** |
| Condition ST * time1 | 3.99 | 1.78 | 2.24 | 206 | 0.03 |
| Condition ST * time2 | 2.87 | 2.00 | 1.44 | 206 | 0.15 |
| Time 1 * RSES | 0.35 | 0.19 | 1.88 | 206 | 0.06 |
| Time 2 * RSES | **0.61** | 0.21 | 2.94 | 206 | **<0.01** |

Note. Cognitive Behavioral Therapy (CBT) was reference category. ^a^ RSES was mean centered, RSES = Rosenberg Self-Esteem Scale, ST = Schema Therapy.

**Supplementary Tables A6**

Effects of baseline characteristics for the hazard of attrition

**Supplementary Table A6a**

Inventory of depressive symptomatology: Estimated parameters for Cox regression regarding baseline scores

|  | exp.coef. | CI low | CI high | p |
| --- | --- | --- | --- | --- |
| Group schema therapy | 0.39 | 0.24 | 0.63 | <.01 |
| Inventory of depressive symptomatology^a^ | 1.02 | 1.00 | 1.04 | 0.02 |

^a^ Score was mean centered. CI = confidence interval, CI low = 95% lower CI, CI high= 95% higher CI. CTQ = Childhood Trauma Questionnaire.

**Supplementary Table A6b**

CTQ physical neglect: Estimated parameters for Cox regression regarding baseline scores

|  | exp.coef. | CI low | CI high | p |
| --- | --- | --- | --- | --- |
| Group schema therapy | 0.40 | 0.25 | 0.64 | <.01 |
| CTQ physical neglect^a^ | 1.08 | 1.00 | 1.16 | 0.03 |

^a^ Score was mean centered. CI = confidence interval, CI low = 95% lower CI, CI high= 95% higher CI. CTQ = Childhood Trauma Questionnaire.

**Supplementary Table A6c**

CTQ emotional abuse: Estimated parameters for Cox regression regarding baseline scores

|  | exp.coef. | CI low | CI high | p |
| --- | --- | --- | --- | --- |
| Group schema therapy | 0.35 | 0.21 | 0.56 | <.01 |
| CTQ emotional abuse^a^ | 1.08 | 1.04 | 1.13 | <.01 |

^a^ Score was mean centered. CI = confidence interval, CI low = 95% lower CI, CI high= 95% higher CI. CTQ = Childhood Trauma Questionnaire.

**Supplementary Table A6d**

CTQ physical abuse: Estimated parameters for Cox regression regarding baseline scores

|  | exp.coef. | CI low | CI high | p |
| --- | --- | --- | --- | --- |
| Group schema therapy | 0.39 | 0.24 | 0.63 | <.01 |
| CTQ physical abuse^a^ | 1.12 | 1.04 | 1.20 | <.01 |

^a^ Score was mean centered. CI = confidence interval, CI low = 95% lower CI, CI high= 95% higher CI. CTQ = Childhood Trauma Questionnaire.

**Supplementary Table A6e**

Being married/cohabiting: Estimated parameters for Cox regression regarding baseline scores

|  | exp.coef. | CI low | CI high | p |
| --- | --- | --- | --- | --- |
| Group schema therapy | 0.38 | 0.24 | 0.63 | <.01 |
| Being married/cohabiting | 2.16 | 1.31 | 3.58 | <.01 |

CI = confidence interval, CI low = 95% lower CI, CI high= 95% higher CI.

**Supplementary Table A7** Model comparisons for the Liebowitz Social Anxiety Scale, the Avoidant personality disorder severity index and Treatment attrition

|  | **LSAS** | | | **AVPDSI** | | | **Attrition** | |
| --- | --- | --- | --- | --- | --- | --- | --- | --- |
|  | **Model 1:**  **condition x time** | **Model 2:**  **condition x time + predictor** | **Model 3:**  **condition x time**  **+ predictor x time** | **Model 1:**  **condition x time** | **Model 2:**  **condition x time + predictor** | **Model 3:**  **condition x time + predictor x time** | **Model 1:**  **condition** | **Model 2:**  **condition + predictor** |
| **Demographic variables** |  |  |  |  |  |  |  |  |
| Education level |  |  |  |  |  |  |  |  |
| Work status yes/no |  |  |  |  |  |  |  |  |
| Civil status - with partner yes/no |  |  |  |  |  |  |  |  |
| **Clinical variables** |  |  |  |  |  |  |  |  |
| Total number of Axis I disorders |  |  |  |  |  |  |  |  |
| Depressive disorder present yes/no |  |  |  |  |  |  |  |  |
| Psychological treatment last three years |  |  |  |  |  |  |  |  |
| Medication at start treatment yes/no |  |  |  |  |  |  |  |  |
| Avoidant PD traits |  |  |  |  |  |  |  |  |
| Dependent PD traits |  |  |  |  |  |  |  |  |
| Obsessive compulsive PD traits |  |  |  |  |  |  |  |  |
| Borderline PD traits |  |  |  |  |  |  |  |  |
| **Questionnaires** |  |  |  |  |  |  |  |  |
| Emotional neglect (CTQ-SF) |  |  |  |  |  |  |  |  |
| Physical neglect (CTQ-SF) |  |  |  |  |  |  |  |  |
| Emotional abuse (CTQ-SF) |  |  |  |  |  |  |  |  |
| Physical abuse (CTQ-SF) |  |  |  |  |  |  |  |  |
| Sexual abuse (CTQ-SF) |  |  |  |  |  |  |  |  |
| Depressive symptomatology (IDS-SR) |  |  |  |  |  |  |  |  |
| Acceptance and Action Questionnaire (AAQ-II) |  |  |  |  |  |  |  |  |
| Difficulties in Emotion Regulation (DERS) |  |  |  |  |  |  |  |  |
| Rosenberg Self-Esteem Scale (RSES) |  |  |  |  |  |  |  |  |
| SMI (average score) |  |  |  |  |  |  |  |  |
| SMI healthy adult mode |  |  |  |  |  |  |  |  |
| SMI happy child mode |  |  |  |  |  |  |  |  |
| SMI avoidant protector mode |  |  |  |  |  |  |  |  |

Note: For none of the outcomes a three-way interaction of outcome * time * predictor outperformed one of the other models. Shaded cells indicate the best performing model. LSAS = Liebowitz Social Anxiety Scale, AVPDSI = Avoidant personality disorder severity index, PD = personality disorder, CTQ = childhood trauma questionnaire, IDS = inventory of depressive symptomatology, SMI= schema mode inventory.

**Supplementary Tables A8** LSAS, main effect of predictor

**Supplementary Table A8-a** LSAS, main effect of work and/or study

| **Anova** | **numDF** | **F** | **p** |  |  |
| --- | --- | --- | --- | --- | --- |
| Intercept | 1 | 2162.36 | <.01 |  |  |
| Condition | 1 | 7.73 | 0.01 |  |  |
| Time | 5 | 39.67 | <.01 |  |  |
| Work and/or study | 1 | 17.96 | <.01 |  |  |
| Condition * time | 5 | 1.70 | 0.13 |  |  |
| **Fixed effects** | **B** | **SE** | **t** | **df** | **p** |
| Intercept | 90.85 | 3.32 | 27.36 | 742 | <.01 |
| Condition ST | 5.49 | 3.61 | 1.52 | 742 | 0.13 |
| Time 1 | -17.15 | 1.99 | -8.63 | 742 | <.01 |
| Time 2 | -25.07 | 2.83 | -8.85 | 742 | <.01 |
| Time 3 | -25.99 | 3.00 | -8.67 | 742 | <.01 |
| Time 4 | -30.17 | 3.33 | -9.08 | 742 | <.01 |
| Time 5 | -33.44 | 3.79 | -8.82 | 742 | <.01 |
| Work and/or study | -14.15 | 3.32 | -4.26 | 742 | <.01 |
| Condition ST * time 1 | 6.54 | 2.81 | 2.33 | 742 | 0.02 |
| Condition ST * time 2 | 2.29 | 4.01 | 0.57 | 742 | 0.57 |
| Condition ST * time 3 | 2.31 | 4.37 | 0.53 | 742 | 0.60 |
| Condition ST * time 4 | 5.98 | 4.85 | 1.23 | 742 | 0.22 |
| Condition ST * time 5 | 8.06 | 5.53 | 1.46 | 742 | 0.15 |

Note. Cognitive Behavioral Therapy (CBT) was reference category. ST = Schema Therapy.

**Supplementary Table A8-b** LSAS, main effect of education level

| **Anova** | **numDF** | **F** | **p** |  |  |
| --- | --- | --- | --- | --- | --- |
| Intercept | 1 | 2071.58 | <.01 |  |  |
| Condition | 1 | 7.36 | 0.01 |  |  |
| Time | 5 | 39.21 | <.01 |  |  |
| Education level | 3 | 4.27 | 0.01 |  |  |
| Condition * time | 5 | 1.66 | 0.14 |  |  |
| **Fixed effects** | **B** | **SE** | **t** | **df** | **p** |
| Intercept | 96.36 | 6.69 | 14.40 | 740 | <.01 |
| Condition ST | 5.73 | 3.71 | 1.55 | 740 | 0.12 |
| Time 1 | -17.12 | 2.01 | -8.54 | 740 | <.01 |
| Time 2 | -25.04 | 2.82 | -8.89 | 740 | <.01 |
| Time 3 | -25.82 | 3.03 | -8.53 | 740 | <.01 |
| Time 4 | -30.02 | 3.31 | -9.08 | 740 | <.01 |
| Time 5 | -33.38 | 3.84 | -8.69 | 740 | <.01 |
| Medium level | -10.23 | 6.91 | -1.48 | 740 | 0.14 |
| High level | -15.38 | 7.19 | -2.14 | 740 | 0.03 |
| Advanced level | -21.25 | 7.04 | -3.02 | 740 | <.01 |
| Condition ST * time 1 | 6.45 | 2.83 | 2.28 | 740 | 0.02 |
| Condition ST * time 2 | 2.20 | 3.99 | 0.55 | 740 | 0.58 |
| Condition ST * time 3 | 1.98 | 4.42 | 0.45 | 740 | 0.65 |
| Condition ST * time 4 | 5.71 | 4.83 | 1.18 | 740 | 0.24 |
| Condition ST * time 5 | 7.89 | 5.61 | 1.41 | 740 | 0.16 |

Note. Cognitive Behavioral Therapy (CBT) was reference category. ST = Schema Therapy. Medium = MAVO, MBO, High = HAVO/VWO, Advanced = HBO/WO

**Supplementary Table A8-c** LSAS, main effect of number of symptom disorders

| **Anova** | **numDF** | **F** | **p** |  |  |
| --- | --- | --- | --- | --- | --- |
| Intercept | 1 | 2014.80 | <.01 |  |  |
| Condition | 1 | 7.02 | 0.01 |  |  |
| Time | 5 | 38.91 | <.01 |  |  |
| Number of symptoms disorders^a^ | 1 | 4.04 | 0.05 |  |  |
| Condition * time | 5 | 1.66 | 0.14 |  |  |
| **Fixed effects** | **B** | **SE** | **t** | **df** | **p** |
| Intercept | 81.66 | 2.58 | 31.67 | 742 | <.01 |
| Condition ST | 6.74 | 3.71 | 1.82 | 742 | 0.07 |
| Time 1 | -17.08 | 2.00 | -8.56 | 742 | <.01 |
| Time 2 | -24.97 | 2.82 | -8.85 | 742 | <.01 |
| Time 3 | -25.82 | 3.03 | -8.53 | 742 | <.01 |
| Time 4 | -30.00 | 3.34 | -8.97 | 742 | 0.00 |
| Time 5 | -33.32 | 3.86 | -8.64 | 742 | 0.00 |
| Number of symptoms disorders^a^ | 2.84 | 1.42 | 2.01 | 742 | 0.05 |
| Condition ST * time 1 | 6.44 | 2.82 | 2.29 | 742 | 0.02 |
| Condition ST * time 2 | 2.16 | 4.00 | 0.54 | 742 | 0.59 |
| Condition ST * time 3 | 2.06 | 4.42 | 0.47 | 742 | 0.64 |
| Condition ST * time 4 | 5.71 | 4.88 | 1.17 | 742 | 0.24 |
| Condition ST * time 5 | 7.86 | 5.63 | 1.40 | 742 | 0.16 |

Note. Cognitive Behavioral Therapy (CBT) was reference category. ^a^ Score was mean centered. ST = Schema Therapy.

**Supplementary Table A8-d** LSAS, main effect of traits of avoidant personality disorder

| **Anova** | **numDF** | **F** | **p** |  |  |
| --- | --- | --- | --- | --- | --- |
| Intercept | 1 | 2518.93 | 0.00 |  |  |
| Condition | 1 | 8.03 | 0.01 |  |  |
| Time | 5 | 42.00 | 0.00 |  |  |
| AVPD traits ^a^ | 1 | 47.23 | 0.00 |  |  |
| Condition * time | 5 | 1.67 | 0.14 |  |  |
| **Fixed effects** | **B** | **SE** | **t** | **df** | **p** |
| Intercept | 81.96 | 2.33 | 35.15 | 742 | <.01 |
| Condition ST | 6.24 | 3.35 | 1.86 | 742 | 0.06 |
| Time 1 | -17.22 | 2.01 | -8.56 | 742 | <.01 |
| Time 2 | -25.02 | 2.77 | -9.04 | 742 | <.01 |
| Time 3 | -25.76 | 2.95 | -8.73 | 742 | <.01 |
| Time 4 | -30.01 | 3.24 | -9.27 | 742 | <.01 |
| Time 5 | -33.36 | 3.72 | -8.97 | 742 | <.01 |
| Traits of AVPD ^a^ | 10.37 | 1.51 | 6.88 | 742 | <.01 |
| Condition ST * time 1 | 6.52 | 2.84 | 2.30 | 742 | 0.02 |
| Condition ST * time 2 | 2.23 | 3.92 | 0.57 | 742 | 0.57 |
| Condition ST * time 3 | 1.97 | 4.30 | 0.46 | 742 | 0.65 |
| Condition ST * time 4 | 5.68 | 4.72 | 1.20 | 742 | 0.23 |
| Condition ST * time 5 | 7.77 | 5.43 | 1.43 | 742 | 0.15 |

Note. Cognitive Behavioral Therapy (CBT) was reference category. ^a^ Score was mean centered. ST = Schema Therapy, AVPD = avoidant personality disorder.

**Supplementary Table A8-e** LSAS, main effect of traits of dependent personality disorder

| **Anova** | **numDF** | **F** | **p** |  |  |
| --- | --- | --- | --- | --- | --- |
| Intercept | 1 | 2019.05 | <.01 |  |  |
| Condition | 1 | 6.96 | 0.01 |  |  |
| time | 5 | 39.02 | <.01 |  |  |
| Traits of DPD ^a^ | 1 | 4.71 | 0.03 |  |  |
| Condition * time | 5 | 1.65 | 0.15 |  |  |
| **Fixed effects** | **B** | **SE** | **t** | **df** | **p** |
| Intercept | 81.59 | 2.58 | 31.67 | 742 | <.01 |
| Condition ST | 6.88 | 3.70 | 1.86 | 742 | 0.06 |
| Time 1 | -17.12 | 2.00 | -8.54 | 742 | <.01 |
| Time 2 | -25.00 | 2.82 | -8.88 | 742 | <.01 |
| Time 3 | -25.81 | 3.04 | -8.49 | 742 | <.01 |
| Time 4 | -29.98 | 3.32 | -9.03 | 742 | <.01 |
| Time 5 | -33.27 | 3.85 | -8.63 | 742 | <.01 |
| Traits of DPD ^a^ | 3.36 | 1.56 | 2.16 | 742 | 0.03 |
| Condition ST * time 1 | 6.45 | 2.83 | 2.28 | 742 | 0.02 |
| Condition ST * time 2 | 2.19 | 4.00 | 0.55 | 742 | 0.58 |
| Condition ST * time 3 | 2.03 | 4.43 | 0.46 | 742 | 0.65 |
| Condition ST * time 4 | 5.70 | 4.85 | 1.18 | 742 | 0.24 |
| Condition ST * time 5 | 7.80 | 5.63 | 1.39 | 742 | 0.17 |

Note. Cognitive Behavioral Therapy (CBT) was reference category. ^a^ Score was mean centered. ST = Schema Therapy, DPD = dependent personality disorder.

**Supplementary Table A8-f** LSAS, main effect of Inventory of depressive symptomatology

| **Anova** | **numDF** | **F** | **p** |  |  |
| --- | --- | --- | --- | --- | --- |
| Intercept | 1 | 2567.28 | <.01 |  |  |
| Condition | 1 | 7.01 | 0.01 |  |  |
| Time | 5 | 42.22 | <.01 |  |  |
| IDS ^a^ | 1 | 43.89 | <.01 |  |  |
| Condition * time | 5 | 1.72 | 0.13 |  |  |
| **Fixed effects** | **B** | **SE** | **t** | **df** | **p** |
| Intercept | 81.72 | 2.31 | 35.45 | 742 | <.01 |
| Condition ST | 6.03 | 3.32 | 1.82 | 742 | 0.07 |
| Time 1 | -17.31 | 2.01 | -8.62 | 742 | <.01 |
| Time 2 | -25.11 | 2.77 | -9.07 | 742 | <.01 |
| Time 3 | -25.93 | 2.92 | -8.88 | 742 | <.01 |
| Time 4 | -30.03 | 3.25 | -9.24 | 742 | <.01 |
| Time 5 | -33.22 | 3.76 | -8.83 | 742 | <.01 |
| IDS | 0.84 | 0.13 | 6.63 | 742 | <.01 |
| Condition ST * time 1 | 6.63 | 2.83 | 2.34 | 742 | 0.02 |
| Condition ST * time 2 | 2.39 | 3.92 | 0.61 | 742 | 0.54 |
| Condition ST * time 3 | 2.39 | 4.26 | 0.56 | 742 | 0.58 |
| Condition ST * time 4 | 6.04 | 4.74 | 1.28 | 742 | 0.20 |
| Condition ST * time 5 | 8.21 | 5.49 | 1.50 | 742 | 0.14 |

Note. Cognitive Behavioral Therapy (CBT) was reference category. ^a^ Score was mean centered. ST = Schema Therapy, IDS = Inventory of depressive symptomatology.

**Supplementary Table A8-g** LSAS, main effect of Acceptance and Action Questionnaire

| **Anova** | **numDF** | **F** | **p** |  |  |
| --- | --- | --- | --- | --- | --- |
| Intercept | 1 | 2302.95 | <.01 |  |  |
| Condition | 1 | 5.96 | 0.02 |  |  |
| Time | 5 | 41.47 | <.01 |  |  |
| AAQ^a^ | 1 | 32.47 | <.01 |  |  |
| Condition * time | 5 | 2.25 | 0.05 |  |  |
| **Fixed effects** | **B** | **SE** | **t** | **df** | **p** |
| Intercept | 82.87 | 2.42 | 34.22 | 703 | <.01 |
| Condition ST | 3.57 | 3.49 | 1.02 | 703 | 0.31 |
| Time 1 | -17.26 | 2.00 | -8.65 | 703 | <.01 |
| Time 2 | -25.73 | 2.83 | -9.08 | 703 | <.01 |
| Time 3 | -26.78 | 3.03 | -8.83 | 703 | <.01 |
| Time 4 | -32.01 | 3.22 | -9.93 | 703 | <.01 |
| Time 5 | -35.50 | 3.84 | -9.25 | 703 | <.01 |
| AAQ | -1.00 | 0.17 | -5.72 | 703 | <.01 |
| Condition ST * time 1 | 7.74 | 2.82 | 2.74 | 703 | 0.01 |
| Condition ST * time 2 | 3.09 | 4.03 | 0.77 | 703 | 0.44 |
| Condition ST * time 3 | 4.07 | 4.39 | 0.93 | 703 | 0.36 |
| Condition ST * time 4 | 7.33 | 4.70 | 1.56 | 703 | 0.12 |
| Condition ST * time 5 | 9.91 | 5.62 | 1.77 | 703 | 0.08 |

Note. Cognitive Behavioral Therapy (CBT) was reference category. ^a^ Score was mean centered. ST = Schema Therapy, AAQ = Acceptance and Action Questionnaire

**Supplementary Table A8-h** LSAS, main effect of Difficulties in Emotion Regulation Scale

| **Anova** | **numDF** | **F** | **p** |  |  |
| --- | --- | --- | --- | --- | --- |
| Intercept | 1 | 2222.52 | <.01 |  |  |
| Condition | 1 | 6.83 | 0.01 |  |  |
| Time | 5 | 40.27 | <.01 |  |  |
| DERS ^ab^ | 1 | 17.57 | <.01 |  |  |
| Condition * time | 5 | 1.68 | 0.14 |  |  |
| **Fixed effects** | **B** | **SE** | **t** | **df** | **p** |
| Intercept | 81.71 | 2.46 | 33.19 | 742 | <.01 |
| Condition ST | 6.69 | 3.54 | 1.89 | 742 | 0.06 |
| Time 1 | -17.20 | 2.00 | -8.62 | 742 | <.01 |
| Time 2 | -25.03 | 2.80 | -8.94 | 742 | <.01 |
| Time 3 | -25.85 | 3.00 | -8.63 | 742 | <.01 |
| Time 4 | -29.99 | 3.30 | -9.09 | 742 | <.01 |
| Time 5 | -33.26 | 3.83 | -8.70 | 742 | <.01 |
| DERS ^a^ | 0.31 | 0.07 | 4.19 | 742 | <.01 |
| Condition ST * time 1 | 6.50 | 2.81 | 2.31 | 742 | 0.02 |
| Condition ST * time 2 | 2.15 | 3.97 | 0.54 | 742 | 0.59 |
| Condition ST * time 3 | 1.99 | 4.37 | 0.46 | 742 | 0.65 |
| Condition ST * time 4 | 5.60 | 4.81 | 1.16 | 742 | 0.25 |
| Condition ST * time 5 | 7.76 | 5.59 | 1.39 | 742 | 0.17 |

Note: Note. Cognitive Behavioral Therapy (CBT) was reference category. ^a^ Score was mean centered. ST = Schema Therapy, DERS = Difficulties in Emotion Regulation Scale, ^b^ DERS awareness items are excluded.

**Supplementary Table A8-i** LSAS, main effect of Rosenberg Self-Esteem Scale

| **Anova** | **numDF** | **F-value** | **p-value** |  |  |
| --- | --- | --- | --- | --- | --- |
| Intercept | 1 | 2187.54 | <.01 |  |  |
| Condition | 1 | 6.52 | 0.01 |  |  |
| Time | 5 | 40.08 | <.01 |  |  |
| RSES ^a^ | 1 | 13.02 | <.01 |  |  |
| Condition * time | 5 | 1.70 | 0.13 |  |  |
| **Fixed effects** | **B** | **SE** | **t** | **df** | **p** |
| Intercept | 81.69 | 2.48 | 32.99 | 742 | <.01 |
| conditionST | 6.92 | 3.56 | 1.94 | 742 | 0.05 |
| Time 1 | -17.21 | 1.98 | -8.68 | 742 | <.01 |
| Time 2 | -25.06 | 2.80 | -8.95 | 742 | <.01 |
| Time 3 | -25.94 | 2.99 | -8.67 | 742 | <.01 |
| Time 4 | -30.13 | 3.34 | -9.04 | 742 | <.01 |
| Time 5 | -33.37 | 3.85 | -8.67 | 742 | <.01 |
| RSES ^a^ | -1.27 | 0.35 | -3.60 | 742 | <.01 |
| Condition ST * time 1 | 6.50 | 2.80 | 2.32 | 742 | 0.02 |
| Condition ST * time 2 | 2.16 | 3.97 | 0.54 | 742 | 0.59 |
| Condition ST * time 3 | 2.10 | 4.36 | 0.48 | 742 | 0.63 |
| Condition ST * time 4 | 5.76 | 4.87 | 1.18 | 742 | 0.24 |
| Condition ST * time 5 | 7.81 | 5.62 | 1.39 | 742 | 0.17 |

Note. Cognitive Behavioral Therapy (CBT) was reference category. ^a^ Score was mean centered. ST = Schema Therapy, RSES = Rosenberg Self-Esteem Scale

**Supplementary Table A8-j** LSAS, main effect of Schema mode inventory - average score

| **Anova** | **numDF** | **F** | **p** |  |  |
| --- | --- | --- | --- | --- | --- |
| Intercept | 1 | 2380.66 | <.01 |  |  |
| Condition | 1 | 6.92 | 0.01 |  |  |
| Time | 5 | 41.20 | <.01 |  |  |
| SMI-AV^a^ | 1 | 30.67 | <.01 |  |  |
| Condition * time | 5 | 1.68 | 0.14 |  |  |
| **Fixed effects** | **B** | **SE** | **t** | **df** | **p** |
| Intercept | 81.54 | 2.39 | 34.12 | 742 | <.01 |
| Condition ST | 6.95 | 3.44 | 2.02 | 742 | 0.04 |
| Time 1 | -17.25 | 2.01 | -8.58 | 742 | <.01 |
| Time 2 | -25.02 | 2.80 | -8.95 | 742 | <.01 |
| Time 3 | -25.78 | 2.97 | -8.69 | 742 | <.01 |
| Time 4 | -29.92 | 3.24 | -9.24 | 742 | <.01 |
| Time 5 | -33.15 | 3.79 | -8.75 | 742 | <.01 |
| SMI-AV | 17.62 | 3.19 | 5.53 | 742 | <.01 |
| Condition ST * time 1 | 6.51 | 2.83 | 2.30 | 742 | 0.02 |
| Condition ST * time 2 | 2.09 | 3.96 | 0.53 | 742 | 0.60 |
| Condition ST * time 3 | 1.88 | 4.32 | 0.43 | 742 | 0.67 |
| Condition ST * time 4 | 5.50 | 4.72 | 1.16 | 742 | 0.25 |
| Condition ST * time 5 | 7.65 | 5.53 | 1.38 | 742 | 0.17 |

Note. Cognitive Behavioral Therapy (CBT) was reference category. ^a^ Score was mean centered. ST = Schema Therapy, SMI-AV = Schema mode inventory - average score

**Supplementary Table A8-k** LSAS, main effect of Schema mode inventory - Healthy Adult

| **Anova** | **numDF** | **F** | **p** |  |  |
| --- | --- | --- | --- | --- | --- |
| Intercept | 1 | 2234.07 | <.01 |  |  |
| Condition | 1 | 6.67 | 0.01 |  |  |
| Time | 5 | 40.31 | <.01 |  |  |
| SMI-HA | 1 | 18.75 | <.01 |  |  |
| Condition * time | 5 | 1.68 | 0.14 |  |  |
| **Fixed effects** | **B** | **SE** | **t** | **df** | **p** |
| Intercept | 81.68 | 2.46 | 33.22 | 742 | <.01 |
| Condition ST | 6.73 | 3.54 | 1.90 | 742 | 0.06 |
| Time 1 | -17.23 | 2.01 | -8.56 | 742 | <.01 |
| Time 2 | -25.01 | 2.80 | -8.94 | 742 | <.01 |
| Time 3 | -25.82 | 2.98 | -8.67 | 742 | <.01 |
| Time 4 | -29.95 | 3.27 | -9.15 | 742 | <.01 |
| Time 5 | -33.19 | 3.84 | -8.64 | 742 | <.01 |
| SMI-HA | -10.73 | 2.48 | -4.33 | 742 | <.01 |
| Condition ST * time 1 | 6.49 | 2.84 | 2.29 | 742 | 0.02 |
| Condition ST * time 2 | 2.06 | 3.96 | 0.52 | 742 | 0.60 |
| Condition ST * time 3 | 1.91 | 4.34 | 0.44 | 742 | 0.66 |
| Condition ST * time 4 | 5.57 | 4.78 | 1.17 | 742 | 0.24 |
| Condition ST * time 5 | 7.79 | 5.61 | 1.39 | 742 | 0.17 |

Note. Cognitive Behavioral Therapy (CBT) was reference category. ^a^ Score was mean centered. ST = Schema Therapy, SMI-HA = Schema mode inventory – Healthy Adult

**Supplementary Table A8-l** LSAS, main effect of Schema mode inventory - Happy Child

| **Anova** | **numDF** | **F** | **p** |  |  |
| --- | --- | --- | --- | --- | --- |
| Intercept | 1 | 2326.06 | <.01 |  |  |
| Condition | 1 | 7.04 | 0.01 |  |  |
| Time | 5 | 40.82 | <.01 |  |  |
| SMI-HC | 1 | 26.10 | <.01 |  |  |
| Condition * time | 5 | 1.69 | 0.14 |  |  |
| **Fixed effects** | **B** | **SE** | **t** | **df** | **p** |
| Intercept | 81.95 | 2.41 | 33.96 | 742 | <.01 |
| Condition ST | 6.01 | 3.47 | 1.73 | 742 | 0.08 |
| Time 1 | -17.16 | 1.99 | -8.64 | 742 | <.01 |
| Time 2 | -24.94 | 2.81 | -8.88 | 742 | <.01 |
| Time 3 | -25.67 | 2.97 | -8.64 | 742 | <.01 |
| Time 4 | -29.89 | 3.29 | -9.10 | 742 | <.01 |
| Time 5 | -33.10 | 3.79 | -8.73 | 742 | <.01 |
| SMI-HC | -12.45 | 2.44 | -5.10 | 742 | <.01 |
| Condition ST * time 1 | 6.41 | 2.80 | 2.29 | 742 | 0.02 |
| Condition ST * time 2 | 1.95 | 3.98 | 0.49 | 742 | 0.62 |
| Condition ST * time 3 | 1.76 | 4.33 | 0.41 | 742 | 0.69 |
| Condition ST * time 4 | 5.46 | 4.79 | 1.14 | 742 | 0.26 |
| Condition ST * time 5 | 7.63 | 5.54 | 1.38 | 742 | 0.17 |

Note. Cognitive Behavioral Therapy (CBT) was reference category. ^a^ Score was mean centered. ST = Schema Therapy, SMI-HC = Schema mode inventory – Happy Child

**Supplementary Tables A9** AVPDSI, main effect of predictor

**Supplementary Table A9-a** AVPDSI, Main effect of being married/cohabiting

| **Anova** | **numDF** | **denDF** | **F** | **p** |  |
| --- | --- | --- | --- | --- | --- |
| Intercept | 1 | 208 | 4467.68 | <.01 |  |
| Condition | 1 | 143 | 0.47 | 0.50 |  |
| Time | 2 | 208 | 44.85 | <.01 |  |
| Being married/cohabiting | 1 | 143 | 6.29 | 0.01 |  |
| Condition * time | 2 | 208 | 2.41 | 0.09 |  |
| **Fixed effects** | **Value** | **SE** | **t** | **DF** | **p** |
| Intercept | 54.32 | 1.18 | 46 | 208 | <.01 |
| Condition ST | 0.11 | 1.61 | 0.07 | 143 | 0.94 |
| Time 1 | -8.69 | 1.25 | -6.971 | 208 | <.01 |
| Time 2 | -10.81 | 1.43 | -7.553 | 208 | <.01 |
| Being married/cohabiting | -4.81 | 1.94 | -2.477 | 143 | 0.01 |
| Condition ST * time 1 | 3.93 | 1.79 | 2.19 | 208 | 0.03 |
| Condition ST * time 2 | 2.66 | 2.05 | 1.296 | 208 | 0.20 |

Note. Cognitive Behavioral Therapy (CBT) was reference category. ST = Schema Therapy.

**Supplementary Table A9-b** AVPDSI, Main effect of number of symptom disorders

| **Anova** | **numDF** | **denDF** | **F** | **p** |  |
| --- | --- | --- | --- | --- | --- |
| Intercept | 1 | 208 | 4456.55 | <.01 |  |
| Condition | 1 | 143 | 0.41 | 0.52 |  |
| Time | 2 | 208 | 44.85 | <.01 |  |
| Number of symptom disorders^a^ | 1 | 143 | 5.69 | 0.02 |  |
| Condition * time | 2 | 208 | 2.48 | 0.09 |  |
| **Fixed effects** | **Value** | **SE** | **t** | **DF** | **p** |
| Intercept | 53.55 | 1.12 | 47.68 | 208 | <.01 |
| Condition ST | -0.20 | 1.61 | -0.13 | 143 | 0.90 |
| Time 1 | -8.67 | 1.25 | -6.95 | 208 | <.01 |
| Time 2 | -10.79 | 1.43 | -7.54 | 208 | <.01 |
| Number of symptom disorders* | 1.57 | 0.66 | 2.39 | 143 | 0.02 |
| Condition ST * time 1 | 3.99 | 1.79 | 2.22 | 208 | 0.03 |
| Condition ST * time 2 | 2.70 | 2.05 | 1.32 | 208 | 0.19 |

Note. Cognitive Behavioral Therapy (CBT) was reference category. ^a^ Score was mean centered. ST = Schema Therapy.

**Supplementary Table A9-c** AVPDSI, Main effect of traits of avoidant personality disorder

| **Anova** | **numDF** | **denDF** | **F** | **p** |  |
| --- | --- | --- | --- | --- | --- |
| Intercept | 1 | 208 | 5127.47 | <.01 |  |
| Condition | 1 | 143 | 0.46 | 0.50 |  |
| Time | 2 | 208 | 44.15 | <.01 |  |
| AVPD traits ^a^ | 1 | 143 | 27.58 | <.01 |  |
| Condition * time | 2 | 208 | 2.56 | 0.08 |  |
| **Fixed effects** | **Value** | **SE** | **t** | **DF** | **p** |
| Intercept | 53.55 | 1.05 | 50.88 | 208 | <.01 |
| Condition ST | -0.30 | 1.51 | -0.20 | 143 | 0.84 |
| Time 1 | -8.65 | 1.25 | -6.92 | 208 | <.01 |
| Time 2 | -10.77 | 1.43 | -7.51 | 208 | <.01 |
| AVPD traits ^a^ | 3.85 | 0.73 | 5.27 | 143 | <.01 |
| Condition ST * time 1 | 4.06 | 1.80 | 2.26 | 208 | 0.03 |
| Condition ST * time 2 | 2.81 | 2.05 | 1.37 | 208 | 0.17 |

Note. Cognitive Behavioral Therapy (CBT) was reference category. a Score was mean centered. ST = Schema Therapy.

**Supplementary Table A9-d** AVPDSI, Main effect of traits of dependent personality disorder

| **Anova** | **numDF** | **denDF** | **F** | **p** |  |
| --- | --- | --- | --- | --- | --- |
| Intercept | 1 | 208 | 4469.01 | <.01 |  |
| Condition | 1 | 143 | 0.43 | 0.51 |  |
| Time | 2 | 208 | 44.86 | <.01 |  |
| Traits of DPD ^a^ | 1 | 143 | 5.80 | 0.02 |  |
| Condition * time | 2 | 208 | 2.51 | 0.08 |  |
| **Fixed effects** | **Value** | **SE** | **t** | **DF** | **p** |
| Intercept | 53.46 | 1.12 | 47.65 | 208 | <.01 |
| Condition ST | -0.05 | 1.61 | -0.03 | 143 | 0.97 |
| Time 1 | -8.68 | 1.25 | -6.96 | 208 | <.01 |
| Time 2 | -10.79 | 1.43 | -7.53 | 208 | <.01 |
| Traits of DPD ^a^ | 1.73 | 0.71 | 2.42 | 143 | 0.02 |
| Condition ST * time 1 | 4.01 | 1.79 | 2.24 | 208 | 0.03 |
| Condition ST * time 2 | 2.68 | 2.05 | 1.31 | 208 | 0.19 |

Note. Cognitive Behavioral Therapy (CBT) was reference category. ^a^ Score was mean centered. ST = Schema Therapy

**Supplementary Table A9-e** AVPDSI, Main effect of Childhood Trauma Questionnaire - emotional neglect

| **Anova** | **numDF** | **denDF** | **F** | **p** |  |
| --- | --- | --- | --- | --- | --- |
| Intercept | 1 | 208 | 4439.31 | <.01 |  |
| Condition | 1 | 143 | 0.40 | 0.53 |  |
| Time | 2 | 208 | 44.81 | <.01 |  |
| CTQ-emotional neglect^a^ | 1 | 143 | 4.42 | 0.04 |  |
| Condition * time | 2 | 208 | 2.50 | 0.09 |  |
| **Fixed effects** | **Value** | **SE** | **t** | **DF** | **p** |
| Intercept | 53.53 | 1.12 | 47.61 | 208 | <.01 |
| Condition ST | -0.25 | 1.62 | -0.16 | 143 | 0.88 |
| Time 1 | -8.66 | 1.25 | -6.93 | 208 | <.01 |
| Time 2 | -10.77 | 1.43 | -7.52 | 208 | <.01 |
| ACTQ_EN* | 0.31 | 0.15 | 2.12 | 143 | 0.04 |
| Condition ST * time 1 | 4.01 | 1.80 | 2.23 | 208 | 0.03 |
| Condition ST * time 2 | 2.70 | 2.05 | 1.32 | 208 | 0.19 |

Note. Cognitive Behavioral Therapy (CBT) was reference category. ^a^ Score was mean centered. ST = Schema Therapy, CTQ = childhood trauma questionnaire.

**Supplementary Table A9-f** AVPDSI, Main effect of Childhood Trauma Questionnaire - emotional abuse

| **Anova** | **numDF** | **denDF** | **F** | **p** |  |
| --- | --- | --- | --- | --- | --- |
| Intercept | 1 | 208 | 4570.30 | <.01 |  |
| Condition | 1 | 143 | 0.43 | 0.51 |  |
| Time | 2 | 208 | 44.83 | <.01 |  |
| CTQ emotional abuse* | 1 | 143 | 8.87 | <.01 |  |
| Condition * time | 2 | 208 | 2.49 | 0.09 |  |
| **Fixed effects** | **Value** | **SE** | **t** | **DF** | **p** |
| Intercept | 53.56 | 1.11 | 48.23 | 208 | <.01 |
| Condition ST | -0.51 | 1.60 | -0.32 | 143 | 0.75 |
| Time 1 | -8.61 | 1.25 | -6.90 | 208 | <.01 |
| Time 2 | -10.71 | 1.43 | -7.48 | 208 | <.01 |
| CTQ emotional abuse* | 0.43 | 0.14 | 2.98 | 143 | <.01 |
| Condition ST * time 1 | 4.00 | 1.80 | 2.23 | 208 | 0.03 |
| Condition ST * time 2 | 2.68 | 2.05 | 1.31 | 208 | 0.19 |

Note. Cognitive Behavioral Therapy (CBT) was reference category. a Score was mean centered. ST = Schema Therapy, CTQ = childhood trauma questionnaire.

**Supplementary Table A9-g** AVPDSI, Main effect of Schema mode inventory – average score

| **Anova** | **numDF** | **denDF** | **F** | **p** |  |
| --- | --- | --- | --- | --- | --- |
| Intercept | 1 | 208 | 5605.09 | <.01 |  |
| Condition | 1 | 143 | 0.35 | 0.55 |  |
| Time | 2 | 208 | 45.15 | <.01 |  |
| SMI-AV | 1 | 143 | 42.13 | <.01 |  |
| Condition * time | 2 | 208 | 2.37 | 0.10 |  |
| **Fixed effects** | **Value** | **SE** | **t** | **DF** | **p** |
| Intercept | 53.46 | 1.00 | 53.35 | 208 | <.01 |
| Condition ST | 0.03 | 1.44 | 0.02 | 143 | 0.98 |
| Time 1 | -8.65 | 1.25 | -6.95 | 208 | <.01 |
| Time 2 | -10.78 | 1.43 | -7.52 | 208 | <.01 |
| SMI-AV | 9.07 | 1.40 | 6.47 | 143 | <.01 |
| Condition ST * time 1 | 3.88 | 1.79 | 2.17 | 208 | 0.03 |
| Condition ST * time 2 | 2.51 | 2.06 | 1.22 | 208 | 0.22 |

Note. Cognitive Behavioral Therapy (CBT) was reference category. a Score was mean centered. ST = Schema Therapy, SMI-AV = Schema mode inventory – average score

**Supplementary Table A9-h** AVPDSI, Main effect of Schema mode inventory – avoidant protector

| **Anova** | **numDF** | **denDF** | **F** | **P** |  |
| --- | --- | --- | --- | --- | --- |
| Intercept | 1 | 208 | 5250.31 | <.01 |  |
| Condition | 1 | 143 | 0.38 | 0.54 |  |
| Time | 2 | 208 | 44.93 | <.01 |  |
| SMI-AP^a^ | 1 | 143 | 30.47 | <.01 |  |
| Condition * time | 2 | 208 | 2.53 | 0.08 |  |
| **Fixed effects** | **Value** | **SE** | **t** | **DF** | **p** |
| Intercept | 53.57 | 1.04 | 51.74 | 208 | <.01 |
| Condition ST | 0.16 | 1.49 | 0.11 | 143 | 0.91 |
| Time 1 | -8.77 | 1.25 | -7.03 | 208 | <.01 |
| Time 2 | -10.87 | 1.43 | -7.59 | 208 | <.01 |
| SMI-AP | 5.00 | 0.91 | 5.53 | 143 | <.01 |
| Condition ST * time 1 | 4.02 | 1.79 | 2.24 | 208 | 0.03 |
| Condition ST * time 2 | 2.65 | 2.05 | 1.29 | 208 | 0.20 |

Note. Cognitive Behavioral Therapy (CBT) was reference category. ^a^ Score was mean centered. ST = Schema Therapy, SMI-AP = Schema mode inventory – avoidant protector

**Supplementary Table A9-i** AVPDSI, Main effect of Schema mode inventory – healthy adult

| **Anova** | **numDF** | **denDF** | **F** | **p** |  |
| --- | --- | --- | --- | --- | --- |
| Intercept | 1 | 208 | 5256.51 | <.01 |  |
| Condition | 1 | 143 | 0.32 | 0.57 |  |
| Time | 2 | 208 | 45.05 | <.01 |  |
| SMI-HA* | 1 | 143 | 30.14 | <.01 |  |
| Condition * time | 2 | 208 | 2.42 | 0.09 |  |
| **Fixed effects** | **Value** | **SE** | **t** | **DF** | **p** |
| Intercept | 53.65 | 1.03 | 51.96 | 208 | <.01 |
| Condition ST | -0.32 | 1.48 | -0.22 | 143 | 0.83 |
| Time 1 | -8.70 | 1.25 | -6.96 | 208 | <.01 |
| Time 2 | -10.82 | 1.44 | -7.54 | 208 | <.01 |
| SMI-HA* | -6.07 | 1.11 | -5.48 | 143 | <.01 |
| Condition ST * time 1 | 3.94 | 1.80 | 2.19 | 208 | 0.03 |
| Condition ST * time 2 | 2.58 | 2.06 | 1.25 | 208 | 0.21 |

Note. Cognitive Behavioral Therapy (CBT) was reference category. ^a^ Score was mean centered. ST = Schema Therapy, SMI-HA = Schema mode inventory - healthy adult

**Supplementary Table A9-j** AVPDSI, Main effect of Schema mode inventory – happy child

| **Anova** | **numDF** | **denDF** | **F** | **p** |  |
| --- | --- | --- | --- | --- | --- |
| Intercept | 1 | 208 | 5403.92 | <.01 |  |
| Condition | 1 | 143 | 0.34 | 0.56 |  |
| Time | 2 | 208 | 45.05 | <.01 |  |
| SMI-HC | 1 | 143 | 35.47 | <.01 |  |
| Condition * time | 2 | 208 | 2.29 | 0.10 |  |
| **Fixed effects** | **Value** | **SE** | **t** | **DF** | **p** |
| Intercept | 53.75 | 1.02 | 52.69 | 208 | <.01 |
| Condition ST | -0.60 | 1.46 | -0.41 | 143 | 0.68 |
| Time 1 | -8.64 | 1.25 | -6.93 | 208 | <.01 |
| Time 2 | -10.78 | 1.43 | -7.52 | 208 | <.01 |
| SMI-HC | -6.40 | 1.08 | -5.92 | 143 | <.01 |
| Condition ST * time 1 | 3.83 | 1.80 | 2.13 | 208 | 0.03 |
| Condition ST * time 2 | 2.51 | 2.06 | 1.22 | 208 | 0.22 |

Note. Cognitive Behavioral Therapy (CBT) was reference category. a Score was mean centered. ST = Schema Therapy, SMI-HC = Schema mode inventory – happy child.

**Supplementary Figure A1**

Multivariate multilevel analyses LSAS: Condition * time + IDS





Note: + IDS is an extension of the condition * time model, therefore graphs are given for both conditions. However, condition * time * outcome was n.s. IDS: Inventory of Depressive Symptoms.

**Supplementary Figure A2**

Multivariate multilevel analyses AVPDSI: Condition * time + SMI-HC





Note: + SMI-HC is an extension of the condition * time model, therefore graphs are given for both conditions. However, condition * time * outcome was n.s. SMI-HC: Schema mode inventory – happy child.

**Supplementary Table A10**

Anova, effect of treatment on putative mediating variables: DERS, RSES, AAQ, SMI and SMI scales

| **Measure** | | **numDF** | **denDF**^a^ | **F** | **p** |
| --- | --- | --- | --- | --- | --- |
| **AAQ** | |  |  |  |  |
|  | Intercept | 1 | 727 | 3428.93 | <.01 |
|  | Condition | 1 | 727 | 3.91 | 0.05 |
|  | Time | 5 | 727 | 13.91 | <.01 |
|  | Condition x time | 5 | 727 | 1.43 | 0.21 |
| **DERS** | |  |  |  |  |
|  | Intercept | 1 | 735 | 3114.91 | <.01 |
|  | Condition | 1 | 735 | 0.89 | 0.35 |
|  | Time | 5 | 735 | 15.66 | <.01 |
|  | Condition x time | 5 | 735 | 0.27 | 0.93 |
| **RSES** | |  |  |  |  |
|  | Intercept | 1 | 740 | 1490.22 | <.01 |
|  | Condition | 1 | 740 | 2.13 | 0.14 |
|  | Time | 5 | 740 | 13.52 | <.01 |
|  | Condition x time | 5 | 740 | 1.32 | 0.26 |
| **SMI scale and subscales** | |  |  |  |  |
| **-** | **SMI** |  |  |  |  |
|  | Intercept | 1 | 733 | 5986.19 | <.01 |
|  | Condition | 1 | 733 | 0.62 | 0.43 |
|  | Time | 5 | 733 | 12.61 | <.01 |
|  | Condition x time | 5 | 733 | 1.31 | 0.26 |
| - | **SMI-HA** |  |  |  |  |
|  | Intercept | 1 | 733 | 4386.38 | <.01 |
|  | Condition | 1 | 733 | 0.77 | 0.38 |
|  | Time | 5 | 733 | 10.42 | <.01 |
|  | Condition x time | 5 | 733 | 1.25 | 0.28 |
| **-** | **SMI-HC** |  |  |  |  |
|  | Intercept | 1 | 733 | 3874.19 | <.01 |
|  | Condition | 1 | 733 | 1.88 | 0.17 |
|  | Time | 5 | 733 | 11.80 | <.01 |
|  | Condition x time | 5 | 733 | 0.83 | 0.53 |
| - | **SMI-AP** |  |  |  |  |
|  | Intercept | 1 | 733 | 4047.77 | <.01 |
|  | Condition | 1 | 733 | 1.05 | 0.30 |
|  | Time | 5 | 733 | 23.72 | <.01 |
|  | Condition x time | 5 | 733 | 0.99 | 0.42 |

Note. ^a^ analyzed using covariance pattern models. AAQ = Acceptance and Action Questionnaire, DERS = Difficulties in Emotion Regulation Scale, RSES = Rosenberg Self-Esteem Scale, SMI = schema mode inventory, SMI-HA = SMI healthy adult mode, SMI-HC = SMI happy child mode, SMI-AP = SMI avoidant protector mode.

**Supplementary Table A11**

Estimated means and within-group effect sizes AAQ, DERS, RSES and SMI per condition, intention-to-treat sample.

| **Measure** | | **T0** | **T1** | **T2** | **T3** | **T4** | **T5** | **T2** | **T5** |
| --- | --- | --- | --- | --- | --- | --- | --- | --- | --- |
|  |  | **EM [CI]** | **EM [CI]** | **EM [CI]** | **EM [CI]** | **EM [CI]** | **EM [CI]** | **d^w^ [CI]** | **d^w^[CI]** |
| **AAQ** | |  |  |  |  |  |  |  |  |
|  | GCBT | 36.0 [33.6;38.4] | 38.4 [35.9;40.9] | 42.0 [39.4;44.6] | 42.1 [39.6;44.7] | 42.1 [39.6;44.7] | 44.5 [41.9;47.2] | 0.67 [0.41;0.92] | 0.94 [0.66;1.22] |
|  | GST | 33.3 [30.8;35.8] | 33.7 [31.2;36.3] | 39.4 [36.9;42.0] | 40.2 [37.5;42.8] | 42.0 [39.3;44.6] | 41.5 [38.7;44.3] | 0.67 [0.41;0.94] | 0.91 [0.62;1.19] |
| **DERS** | |  |  |  |  |  |  |  |  |
|  | GCBT | 90.8 [85.8;95.8) | 85.0 [79.8-90.2] | 77.7 [72.3;83.1] | 75.7 [70.3;81.1] | 72.8 [67.3;78.2] | 71.8 [66.2;77.4] | -0.60 [-0.84;-0.36] | -0.87 [-1.13;-0.61] |
|  | GST | 92.2 [87.0;97.3] | 88.9 [83.6;94.2] | 80.5 [75.1;86.0] | 80.1 [74.6;85.7] | 76.4 [70.8;82.1] | 75.4 [69.5;81.3] | -0.53 [-0.78;-0.29] | -0.77 [-1.03;-0.51] |
| **RSES** | |  |  |  |  |  |  |  |  |
|  | GCBT | 11.3 [10.0;12.5] | 12.8 [11.5-14.1] | 14.6 [13.3-16.0] | 14.6 [13.3;15.9] | 15.3 [14.0-16.6] | 16.2 [14.9-17.6] | 0.73 [0.48;0.98] | 1.08 [0.79;1.36] |
|  | GST | 11.2 [9.9;12.4] | 11.4 [10.1;12.7] | 14.1 [12.8;15.4] | 13.3 [11.9;14.6] | 14.0 [12.6;15.4] | 14.2 [12.7;15.6] | 0.63 [0.38;0.88] | 0.64 [0.39;0.90] |
| **SMI** | |  |  |  |  |  |  |  |  |
|  | GCBT | 3.15 [3.01;3.28] | 3.01 [2.87;3.15] | 2.84 [2.70;2.98] | 2.77 [2.63;2.91] | 2.76 [2.61;2.90] | 2.65 [2.50;2.80] | -0.62 [-0.87; 0.38] | -1.01 [-1.29;-0.73] |
|  | GST | 3.15[3.02;3.29] | 3.12[2.98;3.26] | 2.90[2.76;3.05] | 2.93[2.78;3.08] | 2.90[2.76;3.05] | 2.86[2.70;3.02] | -0.50 [-0.75;-0.26] | -0.59 [-0.84;-0.34] |
| **SMI-HC** | |  |  |  |  |  |  |  |  |
|  | GCBT | 2.83 [2.68;2.98] | 3.08 [2.91;3.26] | 3.26 [3.06;3.46] | 3.33 [3.13;3.54] | 3.34 [3.15;3.53] | 3.47 [3.23;3.70] | 0.66 [0.41; 0.91] | 0.98 [0.70; 1.25] |
|  | GST | 2.75 [2.59;2.90] | 2.85 [2.67;3.02] | 3.14 [2.94;3.34] | 3.11 [2.90;3.32] | 3.15 [2.95;3.35] | 3.26 [3.01;3.50] | 0.60 [0.35; 0.85] | 0.78 [0.52; 1.04] |
| **SMI-HA** | |  |  |  |  |  |  |  |  |
|  | GCBT | 2.98 [2.83;3.14] | 3.20 [3.03;3.36] | 3.37 [3.17;3.56] | 3.47 [3.28;3.67] | 3.45 [3.25;3.65] | 3.58 [3.36;3.80] | 0.59 [0.35;0.83] | 0.92 [0.65;1.19] |
|  | GST | 2.96 [2.80;3.12] | 3.06 [2.89;3.23] | 3.34 [3.14;3.53] | 3.27 [3.07;3.47] | 3.39 [3.18;3.59] | 3.41 [3.18;3.64] | 0.58 [0.33;0.83] | 0.69 [0.43;0.94] |
| **SMI-AP** | |  |  |  |  |  |  |  |  |
|  | GCBT | 4.26 [4.06;4.46] | 3.85 [3.64;4.06] | 3.51 [3.29;3.73] | 3.47 [[3.25;3.69] | 3.40 [3.18;3.62] | 3.28 [3.06;3.51] | -0.96 [-1.23;-0.68] | -1.25 [-1.55;-0.95] |
|  | GST | 4.25 [4.04;4.45] | 3.93 [3.72;4.14] | 3.57 [3.35;3.79] | 3.65 [3.42;3.87] | 3.58 [3.35;3.81] | 3.63 [3.39;3.87] | -0.86 [-1.13;-0.59] | -0.78 [-1.05; -0.52] |

Note: Measurements: T0=baseline, T2= post-treatment, T5= 12-month follow-up. EM=estimated mean, CI= 95% confidence interval, d^w^=effect size within= post-measurement minus pre-measurement, divided by the standard deviation of the pre-measurement. AAQ = Acceptance and Action Questionnaire, DERS = Difficulties in Emotion Regulation Scale, RSES = Rosenberg Self-Esteem Scale, SMI = schema mode inventory, SMI-HC = SMI happy child mode, SMI-HA = SMI healthy adult mode, SMI-AP = SMI avoidant protector mode. GCBT = group cognitive behavioral therapy; GST = group schema therapy.

**Supplementary Table A12** *Outcomes of cross-lagged panel models examining the temporal and mediational relationships of candidate mechanisms of change and social anxiety symptom severity.*

|  | **AAQ** | **DERS** | **RSES** | **SMI^a^** | **SMI-HC** | **SMI-HA** | **SMI-AP** |
| --- | --- | --- | --- | --- | --- | --- | --- |
| **Model fit** |  |  |  |  |  |  |  |
| CFI | .992 | 1.000 | .970 | .998 | 1.000 | .989 | 1.000 |
| SRMR | .013 | .010 | .018 | .010 | .010 | .018 | .012 |
|  |  |  |  |  |  |  |  |
| **Effect of condition** |  |  |  |  |  |  |  |
| anx0 on cond | .137 | .137 | .137 | .137 | .137 | .137 | .137 |
| anx1 on cond | .163 ** | .162 ** | .163 ** | .162** | .165 *** | .166 ** | .156 ** |
| an2 on cond | -.028 | -.016 | -.018 | -.014 | -.028 | -.023 | .013 |
| m0 on cond | -.125 | .012 | .007 | -.017 | -.048 | -.011 | -.021 |
| m1 on cond | -.141 | .083 | -.112 | .092 | -.135 | -.095 | .040 |
| m2 on cond | .018 | -.029 | .059 | -.029 | .049 | .090 | -.031 |
|  |  |  |  |  |  |  |  |
| **Cross-sectional associations** | |  |  |  |  |  |  |
| anx0 with m0 | -.428 *** | .341 *** | -.359 *** | .355 *** | -.409 *** | -.338 *** | .631 *** |
| anx1 with m1 | -.395 *** | .366 *** | -.318 *** | .437 *** | -.334 *** | -.309 ** | .541 *** |
| anx2 with m2 | -.484 *** | .491 *** | -.553 *** | .559*** | -.523 *** | -.576 *** | .686 *** |
|  |  |  |  |  |  |  |  |
| **Autoregressive paths** |  |  |  |  |  |  |  |
| anx1 on anx0 | .677 *** | .685 *** | .692 *** | .695 *** | .686 *** | .701 *** | .726 *** |
| anx2 on anx1 | .716 *** | .719 *** | .697 *** | .705 *** | .736 *** | .724 *** | .648 *** |
| m1 on m0 | .610 *** | .633 *** | .556 *** | .728 *** | .583 *** | .590 *** | .570 *** |
| m2 on m1 | .666*** | .668 *** | .679 *** | .752*** | .574 *** | .651 *** | .634 *** |
|  |  |  |  |  |  |  |  |
| **Cross-lagged effects** |  |  |  |  |  |  |  |
| anx1 on m0 | -.001 | -.022 | .041 | -.049 | .021 | .071 | -.078 |
| anx2 on m1 | -.135 * | .139 * | -.154 | .171 * | -.082 | -.130 * | .184 * |
| m1 on anx0 | .059 | -.027 | -.164 | .010 | -.050 | -.050 | .143 |
| m2 on anx1 | -.078 | .160 * | -.067 | .074 | -.177 * | -.112 | .148 |
|  |  |  |  |  |  |  |  |
| **Indirect mediation effect** |  |  |  |  |  |  |  |
| cond to anx2 via m1 | .019 (-.008; .046) | .012 (-.013; .036) | .017 (-.012; .047) | .016 (-.009; .041) | .011 (-.010; .032) | .012 (-.010; .034) | .007 (-.019-.034) |
| cond to m2 via anx1 | -.013(-.043; .018) | .026 (-.002; .050) | -.011 (-.043; .021) | .012 (-.017; .041) | -.029 (-.065; .006) | -.019 (-.049; .011) | .023 (-.018-.061) |

*Note. * p<.05, **p<.01, ***p<.001; ^a^ average SMI score. Abbreviations: anx=social anxiety symptom severity, m=candidate mediator, cond=condition, CFI=comparative fit index, SRMR=standardized root mean squared residual, AAQ= Acceptance and Action Questionnaire-II, DERS= Difficulties in Emotion Regulation Scale, RSES= Rosenberg Self-Esteem Scale, SMI= Schema Mode Inventory, HC=healthy child mode, HA=healthy adult mode, AP=avoidant protector mode.* CLPM analyses: free parameters 29., 0-model 4 df, CLPM model 21 df.

**Supplementary Table A13** *Per-protocol sample:* *Outcomes of random- intercept cross-lagged panel models examining the temporal and mediational relationships of candidate mechanisms of change and social anxiety symptom severity.*

|  | **AAQ** | **DERS** | **RSES** | **SMI^a^** | **SMI-HC** | **SMI-HA** | **SMI-AP** |
| --- | --- | --- | --- | --- | --- | --- | --- |
| **Model fit** |  |  |  |  |  |  |  |
| CFI | 1.000 | 1.000 | .995 | 1.000 | .993 | .996 | .993 |
| SRMR | .002 | .001 | .030 | .006 | .025 | .020 | .024 |
|  |  |  |  |  |  |  |  |
| **Association between random intercepts** | | |  |  |  |  |  |
| RI anx with RI m | -.546 | .593 | -.360 | .635 | -.239 | -.554 | .698 |
|  |  |  |  |  |  |  |  |
| **Effect of condition** | |  |  |  |  |  |  |
| anx0 on cond | .148 | .092 | .159 | .084 | .069 | .114 | .129 |
| anx1 on cond | .405 | .348 | .306 | .289 | .282 | .371 | .333 |
| an2 on cond | .113 | .188 | .078 | .168 | .178 | .190 | .187 |
| m0 on cond | -.165 | .168 | .168 | .002 | -.093 | .136 | -.108 |
| m1 on cond | -.340 | .234 | -.231 | .233 | -.288 | -.157 | .061 |
| m2 on cond | -.082 | -.024 | .078 | .037 | -.219 | .011 | .114 |
|  |  |  |  |  |  |  |  |
| **Cross-sectional associations** | |  |  |  |  |  |  |
| anx0 with m0 | -.370 | .156 | -.352 | .255 | -.245 | -.137 | .525 |
| anx1 with m1 | -.154 | .027 | -.335 | .213 | -.193 | -.025 | .509 |
| anx2 with m2 | -.541*** | .695 *** | -.659 *** | .525 *** | -.579 *** | -.619*** | .715 *** |
|  |  |  |  |  |  |  |  |
| **Autoregressive paths** | |  |  |  |  |  |  |
| anx1 on anx0 | .228 | .340 | .211 | .465 | .435 | .054 | .368 |
| anx2 on anx1 | .428 | .412 | .335 | .436 | .466 * | .400 | .404 |
| m1 on m0 | .374 | .234 | .113 | .419 | .400 | .245 | .339 |
| m2 on m1 | .489 | .326 | .541 | .622 * | .248 | .586 | .538 |
|  |  |  |  |  |  |  |  |
| **Cross-lagged effects** | |  |  |  |  |  |  |
| anx1 on m0 | .103 | -.169 | .035 | -.196 | -.011 | .247 | .014 |
| anx2 on m1 | -.199 | .213 | -.340 | .325 | -.105 | -.235 | .226 |
| m1 on anx0 | .213 | -.461 | -.122 | -.188 | .053 | .135 | .088 |
| m2 on anx1 | -.028 | .184 | -.003 | -.014 | -.219 | -.083 | .017 |
|  |  |  |  |  |  |  |  |
| **Indirect mediation effects** | |  |  |  |  |  |  |
| cond to anx2 via m1 | .068 (-.276;.411) | .068 (-.262;.397) | .078 (-.188;.344) | .076 (-.201;.352) | .030 (-.077;.158) | .037 (-.215;.289) | .014 (-.539;.567) |
| cond to m2 via anx1 | -.011 (-.287;.264) | .067 (-.147;.281) | -.001 (-.515;.513) | .004 (-.214;.206) | -.062 (-.201;.078) | -.031 (-.298;.237) | .006 (-.556;.567) |

Note. * p<.05, **p<.01, ***p<.001; a average SMI score. Abbreviations: anx=social anxiety symptom severity, m=candidate mediator, cond=condition, CFI=comparative fit index, SRMR = standardized root mean squared residual, AAQ= Acceptance and Action Questionnaire-II, DERS= Difficulties in Emotion Regulation Scale, RSES= Rosenberg Self-Esteem Scale, SMI= Schema Mode Inventory, HC=healthy child mode, HA=healthy adult mode, AP=avoidant protector mode.

**Supplementary Figure A3**

C*ross-lagged panel models examining the temporal and mediational relationships of candidate mechanisms of change and social anxiety symptom severity.*


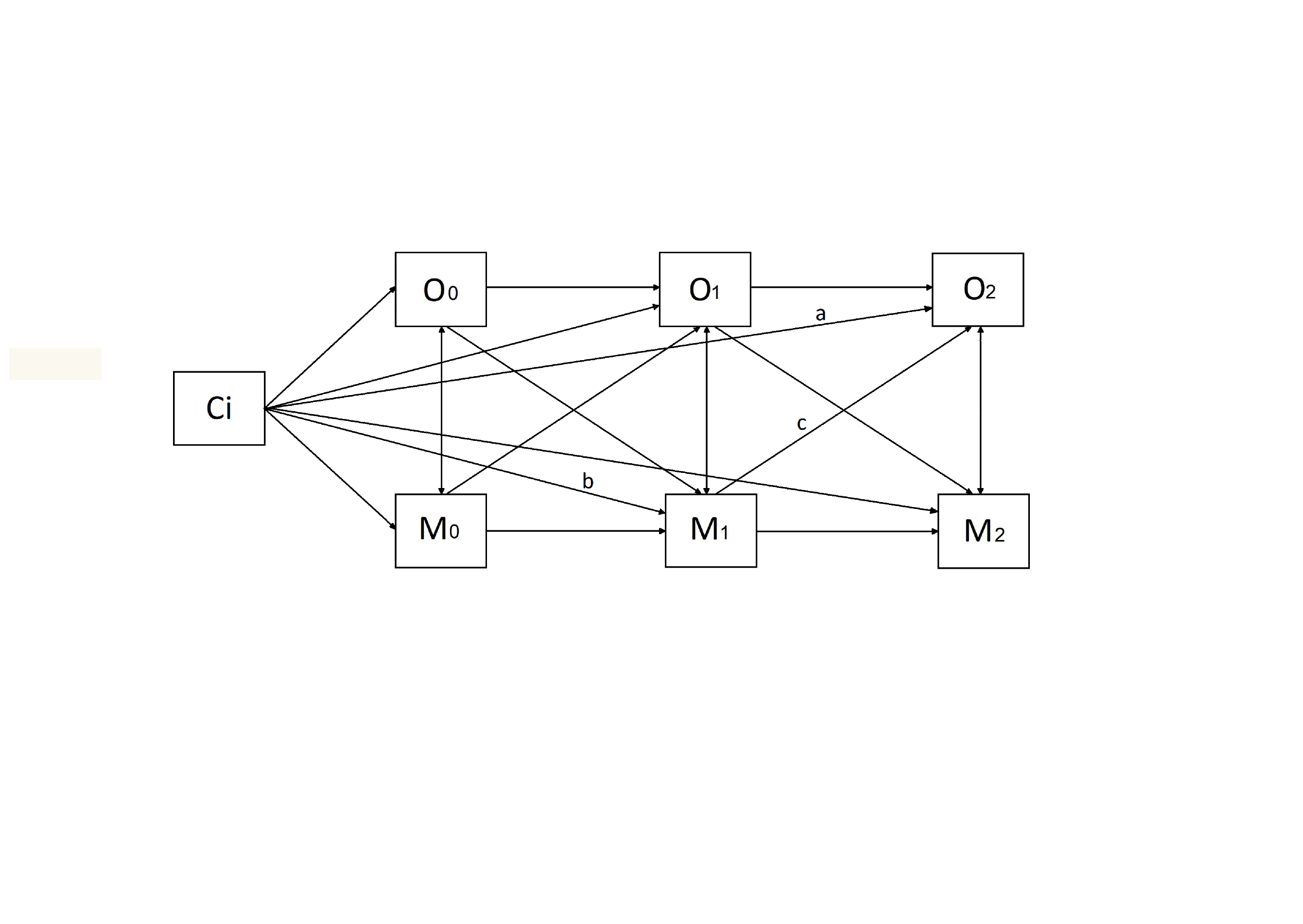

Supplement: Supplementary file 1 — Data S1: Supporting Information. [file CPP-32-e70148-s001.docx]
